# Altered metabolic pathways in clear cell renal cell carcinoma: A meta-analysis and validation study focused on the deregulated genes and their associated networks

**DOI:** 10.18632/oncoscience.13

**Published:** 2014-01-03

**Authors:** Apostolos Zaravinos, Myrtani Pieri, Nikos Mourmouras, Natassa Anastasiadou, Ioanna Zouvani, Dimitris Delakas, Constantinos Deltas

**Affiliations:** ^1^ Molecular Medicine Research Center and Laboratory of Molecular and Medical Genetics, Department of Biological Sciences, University of Cyprus, Nicosia, Cyprus; ^2^ Department of Urology, Asklipieio General Hospital, Athens, Greece; ^3^ Department of Nephrology, Nicosia General Hospital, Nicosia, Cyprus

**Keywords:** clear-cell renal cell carcinoma, oncomine, gene expression, metabolic pathways, pathway analysis, gene networks

## Abstract

Clear cell renal cell carcinoma (ccRCC) is the predominant subtype of renal cell carcinoma (RCC). It is one of the most therapy-resistant carcinomas, responding very poorly or not at all to radiotherapy, hormonal therapy and chemotherapy. A more comprehensive understanding of the deregulated pathways in ccRCC can lead to the development of new therapies and prognostic markers. We performed a meta- analysis of 5 publicly available gene expression datasets and identified a list of co- deregulated genes, for which we performed extensive bioinformatic analysis coupled with experimental validation on the mRNA level. Gene ontology enrichment showed that many proteins are involved in response to hypoxia/oxygen levels and positive regulation of the VEGFR signaling pathway. KEGG analysis revealed that metabolic pathways are mostly altered in ccRCC. Similarly, Ingenuity Pathway Analysis showed that the antigen presentation, inositol metabolism, pentose phosphate, glycolysis/gluconeogenesis and fructose/mannose metabolism pathways are altered in the disease. Cellular growth, proliferation and carbohydrate metabolism, were among the top molecular and cellular functions of the co-deregulated genes. qRT-PCR validated the deregulated expression of several genes in Caki-2 and ACHN cell lines and in a cohort of ccRCC tissues. *NNMT* and *NR3C1* increased expression was evident in ccRCC biopsies from patients using immunohistochemistry. ROC curves evaluated the diagnostic performance of the top deregulated genes in each dataset. We show that metabolic pathways are mostly deregulated in ccRCC and we highlight those being most responsible in its formation. We suggest that these genes are candidate predictive markers of the disease.

## INTRODUCTION

Renal-cell carcinoma (RCC) is the most common kidney neoplasm, comprising 3% of all adult malignancies [1]. Its incidence has increased steadily over the past 20 years in the United States and Europe; 35,000 new cases and 12,000 deaths now occur annually in the United States alone. If detected in early stages, it is potentially curable by surgical resection; however, to date there is no curative treatment for metastatic RCC. Clear cell renal cell carcinoma (ccRCC) represents the most common subtype (83%) of RCC [2]. The most striking phenotypic feature of ccRCC is its clear cell morphology, which has been linked to lipid and glycogen accumulation [3]. Moreover, ccRCC is one of the most therapy-resistant carcinomas, responding very poorly or not at all to radiotherapy, hormonal therapy and chemotherapy. All these facts emphasize the importance of developing early diagnostic markers for this particular RCC subtype.

Meta-analysis consists of statistical techniques to combine results from several studies in order to increase the statistical power and reproducibility compared with any single study [4]. Microarray gene expression profiling has been used in the past by various groups [5-13] to distinguish the various histological subtypes of RCC and consequently to identify novel tumor markers. The general procedure identifies markers in accordance with average differential expression level (fold change) and/or some level of significance as measured by the t-test. All these publicly available microarray expression datasets provide a rich resource for genome-wide information on RCC and provide the ideal opportunity to perform a meta-analysis study using a large number of cases.

The Oncomine database [14] is an online collection of microarray expression data from various cancer-related sources. We hypothesized that a meta-analysis of several gene expression datasets of ccRCC can give a potentially significant list of co-deregulated genes (co-DEGs) in ccRCC, which is important to define pathways in which the genes of interest are involved. To increase the likelihood of revealing truly significant deregulated genes, which should have higher potentials to be used as novel markers for the disease, we analyzed their expression profile over 5 independent studies, greatly increasing the significance of results.

## RESULTS

### Identification of candidate ccRCC markers for network analysis

**Figure 1:**
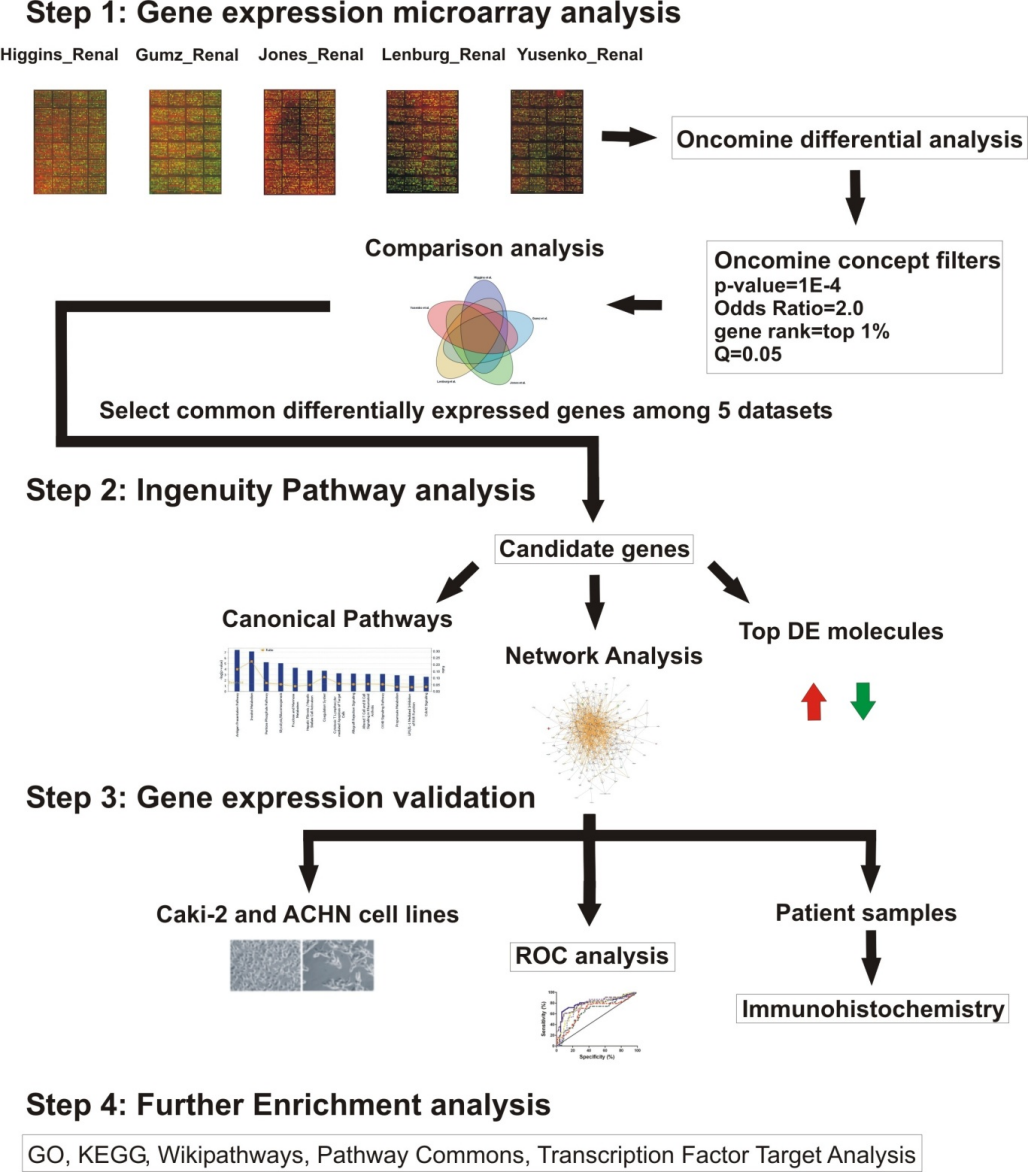
Workflow of the study Initially, five Oncomine microarray datasets were compared and the co-deregulated genes (co-DEGs) among them were retrieved. The co-DEGs were further enquired regarding their use as candidate markers for ccRCC. Next, the canonical pathways in which these co-DEGs are implicated were identified, as well as the networks that they form, and the top deregulated molecules among them. Following, validation of the deregulated expression levels of these genes was performed both in clear cell renal cell carcinoma cell lines, as well as in a cohort of ccRCC patients. Immunohistochemistry was performed in biopsies from the patient cohort for the top deregulated genes. ROC analysis was used to evaluate the discriminatory potential of the candidate biomarker genes. Further enrichment analysis was finally performed for the co-deregulated genes.

**Figure 2:**
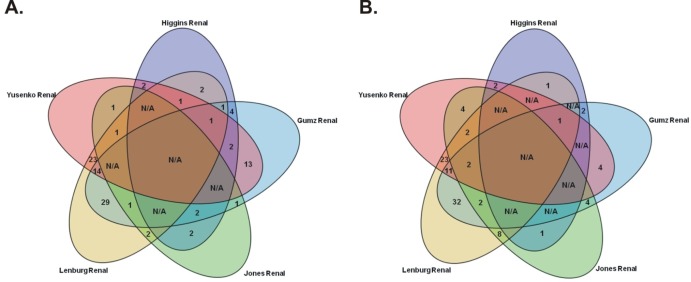
The Venn diagrams depict the co-upregulated (A) and co-downregulated (B) genes in ccRCC vs. their non-tumor kidney tissue, among Oncomine datasets “Higgins Renal”, “Yusenko Renal”, “Lenburg Renal”,”Jones Renal” and “Gumz Renal”

The workflow of our study is summarized in Figure 1. Data mining of five microarray datasets from the Oncomine repository for co- deregulated genes in ccRCC vs. their non-tumor kidney tissue, led to the identification of a list of 93 up- and 76 down-regulated genes, respectively. These genes belonged simultaneously within the top 1% of the up- or down-regulated genes, in at least two datasets, with a p<1E-4 and fold change>2 (Table S1). None of the DEGs belonged simultaneously to the top 1% of the up-regulated genes, in all 5 datasets. The most co- up-regulated gene among 4 datasets was *BTN3A3*. Twenty genes were co-up-regulated among 3 datasets and 93 genes were co-up-regulated between 2 datasets, respectively (Figure 2.A). A similar approach was performed in order to identify the genes that belonged to the top 1% of the down-regulated genes, simultaneously in all 5 datasets. *KCNJ1* and *TMPRSS2* were the top co-down-regulated genes, exhibiting low levels of expression in 4 datasets. Fifteen and 81 genes were co-down-regulated in 3 and 2 datasets, respectively (Figure 2.B).

### Ingenuity Pathway Analysis (IPA) for the top1% of the co-deregulated genes

#### Canonical Pathways

**Figure 3:**
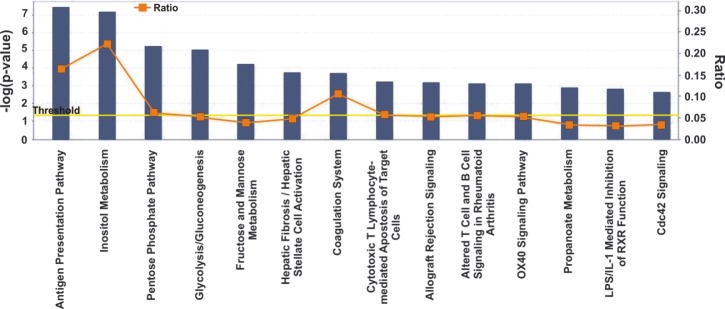
Ingenuity Pathway Analysis (IPA) revealed the top canonical pathways of the top 1% deregulated genes in ccRCC vs. the normal tissue samples, among the 5 Oncomine datasets

Overall, the majority of our deregulated pathways were related to metabolic processes. Specifically, IPA was focused on the following five canonical pathways: The antigen presentation pathway (p=3.71E-08; Ratio, 0.163), containing *HLA-DMB*, *HLA-DPA1*, *HLA- DPB1*, *HLA-DRA*, *PSMBB*, *PSMB9* and *TAPBP* (up-regulated); the inositol metabolism (p=7E-08; Ratio, 0.222), containing *ALDOA*, *ALDOC* (up-regulated), *ALDH6A1* and *ALDOB* (down-regulated); the pentose phosphate pathway (p=5.49E-06; Ratio, 0.062), containing *ALDOA*, *ALDOC*, *PFKP* (up-regulated), *ALDOB* and *FBP1* (down-regulated); glycolysis/gluconeogenesis (p=9.03E-06; Ratio, 0.053), containing *ALDOA*, *ALDOC*, *ENO2*, *LDHA*, *PFKP* (up-regulated), *ALDOB* and *FBP1* (down-regulated); and fructose and mannose metabolism (p=6.02E-05; Ratio, 0.037), containing *ALDOA*, *ALDOC*, *PFKP* (up-regulated), *ALDOB* and *FBP1* (down- regulated) (Figure 3).

#### Correlation between ccRCC and other diseases

To detect any possible correlation between ccRCC and other diseases at the genomic level, we compared the DEGs between ccRCC and other disease gene sets. Comparisons were made with the existing genomic data provided by the software. As expected, ccRCC was highly associated with cancer (p=2.35E-11 - 3.13E-03; 77 molecules); inflammatory response (p=2.86E-11 - 3.00E-03; 44 molecules); renal and urological disease (p=3.95E-10 - 3.00E-03; 32 molecules); reproductive system disease (p=4.39E-09 - 3.00E-03; 46 molecules) and respiratory disease (p=4.69E-09 - 3.02E-03; 31 molecules).

Furthermore, IPA identified that the top co-DEGs among the 5 datasets participate in the following molecular and cellular functions: cell-to-cell signaling and interaction (p=1.56E-09 - 3.00E-03; 42 molecules); cellular function and maintenance (p=3.13E-08 - 2.97E- 03; 48 molecules); molecular transport (p=3.13E-08 - 3.00E-03; 51 molecules); cellular growth and proliferation (p=5.01E-08 - 2.85E-03; 59 molecules) and carbohydrate metabolism (p=7.00E-08 - 2.37E-03; 31 molecules). The top 10 up- and down-regulated molecules in ccRCC vs. the normal tissue are depicted in Table 1.

**Table 1:** Detailed information about the 5 public expression datasets of clear cell renal carcinoma (ccRCC) that were used in the present study

Dataset	Platform	GEO Dataset Accession #	Number of ccRCC samples	Number of normal samples	Citation
Gumz Renal	Affymetrix HU133A & HU133B	GSE6344	20	20	Clin Cancer Res. 2007 Aug 15;13(16):4740-9
Higgins Renal	Affymetrix HU133A	GSE4125	23	3	Am J Pathol. 2003 Mar;162(3):925-32
Jones Renal	Affymetrix HU133A	GSE15641	32	23	Clin Cancer Res. 2005 Aug 15;11(16):5730-9
Lenburg Renal	Affymetrix HU133A & HU133B	GSE781	24	10	BMC Cancer. 2003 Nov 27;3:31
Yusenko Renal	Affymetrix HU133A & HU133B	GSE6280	6	12	Int J Biol Sci. 2009 Jul 29;5(6):517-27

#### Top Transcription Factors

**Figure 4:**
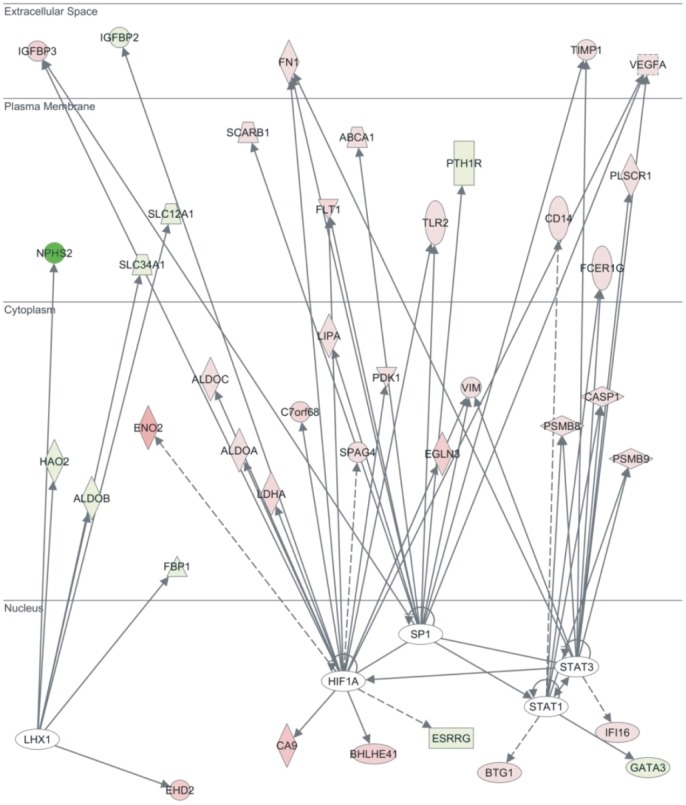
Ingenuity Pathway Analysis (IPA) revealed HIF1A, STAT1, STAT3, SP1 and LHX1 among the top Transcription Factors of the top 1% deregulated genes in ccRCC vs. the normal tissue samples, among the 5 Oncomine datasets

*HIF1A*, *STAT1*, *STAT3*, *SP1* and *LHX1* were the most significant Transcription Factors implicated in the disease, based on the z-score regulation. *HIF1A*, *STAT1*, *STAT3* and *SP1* were predicted to be activated (*HIF1A*: z-score = 2.444; overlap p= 5.68E-12; *STAT1*: z-score = 2.260; overlap p= 1.77E-03; *STAT3*: z-score = 2.125; overlap p= 4.60E-04; *SP1*: z-score = 2.103; overlap p= 9.34E-04). On the other hand, *LHX1* was predicted to be inhibited (z-score = −2.509; overlap p= 2.13E-06). Specifically, 12 out of the 18 *HIF1A* target molecules, were identified to have expression direction consistent with the activation of *HIF1A* (*VEGFA, TLR2, PDK1, LDHA, IGFBP3, FLT1, ESRRG, EGLN3, CA9, C7orf68, ALDOC* and *ALDOA*, activated; *IGFBP2*, inhibited). All *STAT1* target genes exhibited expressional direction consistent with the activation of STAT1 (*PSMB9, PSMB8, FCER1G, CD14, CASP1* and *BTG1*, activated; *GATA3*, inhibited). Six out of the 10 *STAT3* target genes had expressional direction consistent with the activation of *STAT3* (*VIM*, *VEGFA*, *TIMP1*, *PSMB9*, *PSMB8* and *IFI16*, activated). Most *SP1* target genes (7 out of 11) showed expressional direction consistent with the activation of *SP1* (*VEGFA*, *TLR2*, *SCARB1, LIPA, FN1, FLT1* and ABCA1, activated). Also, the majority of the *LHX1* target genes (6 out of 7) had expressional direction consistent with the inhibition of *LHX1 (SLC34A1, SLC12A1, NPHS2, HAO2, FBP1* and *ALDOB*, activated; *EHD2*, activated) (Figure 4).

#### Gene Networks

**Figure 5:**
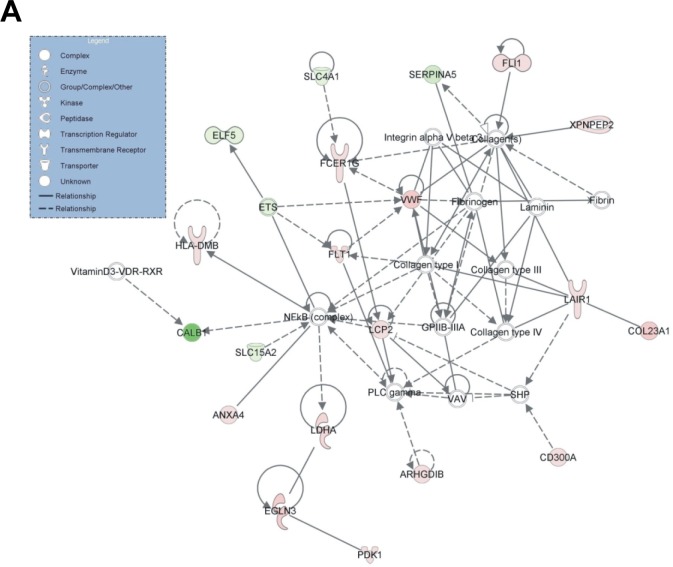

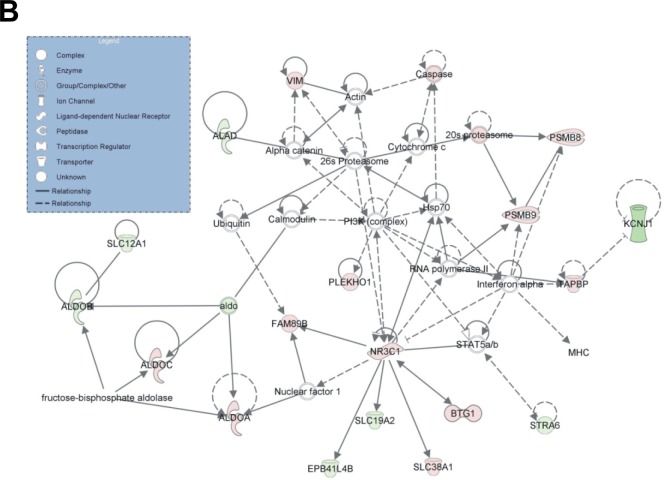

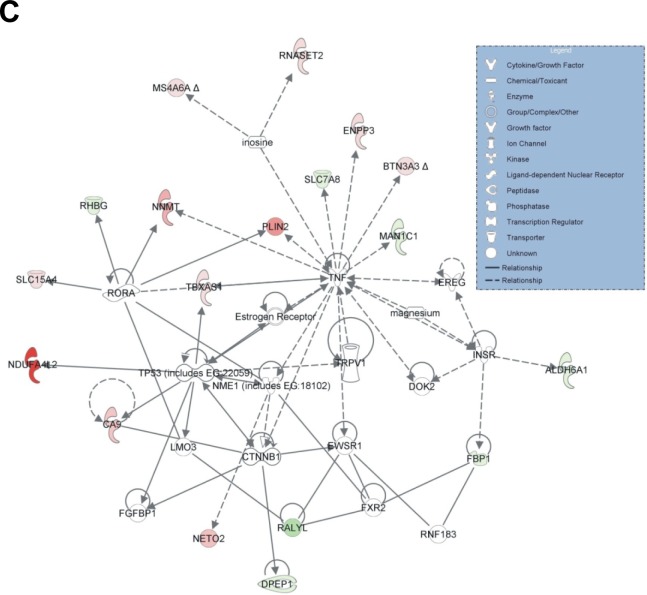

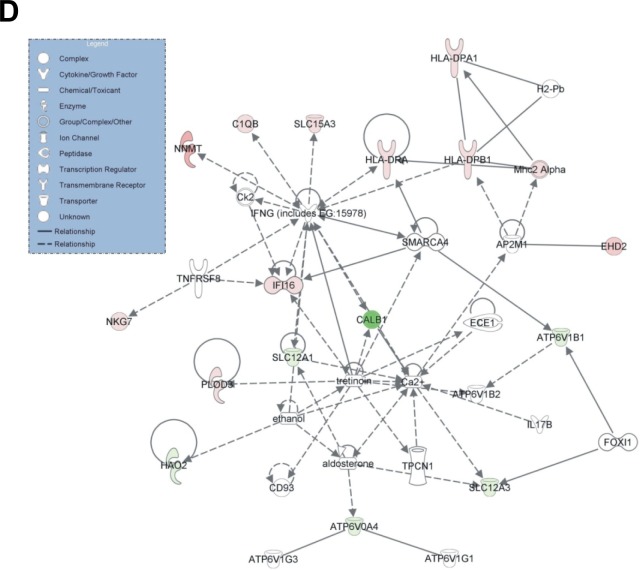

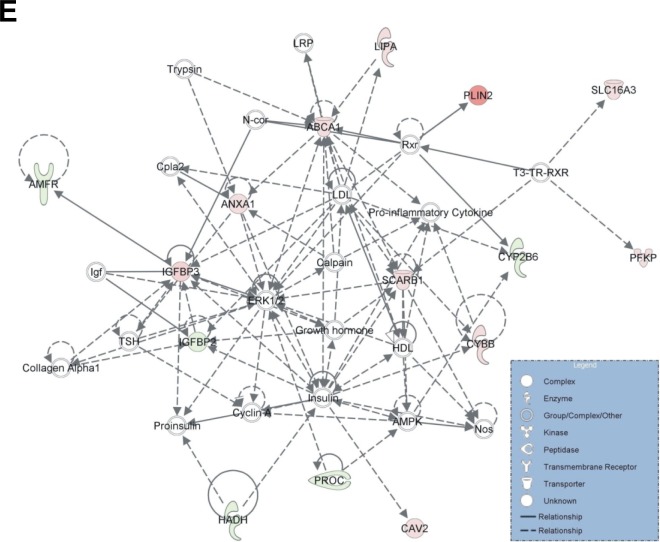
The genes forming the top 5 gene networks as identified by IPA (score>25), participate in: A) Hematological system development and function, cell-to-cell signaling and interaction, reproductive system development and function (score=35); B) Carbohydrate metabolism, cell death, endocrine system disorders (score=33); C) Carbohydrate metabolism, small molecule biochemistry, cellular development (score=30); D) Molecular transport, renal and urological disease, cellular function and maintenance (score=28) and E) Lipid metabolism, small molecule biochemistry, molecular transport (score=26)

**Figure 6:**
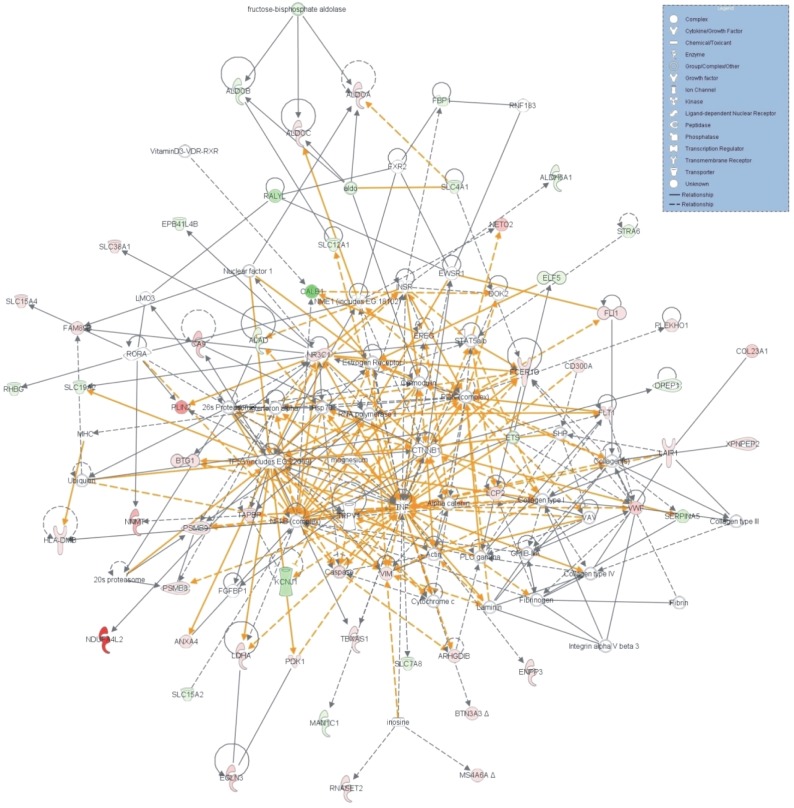
Merge of the top 3 gene networks as revealed by IPA

IPA identified 10 gene networks, with scores ranging from 18 to 35, of which the top 5 were associated with: 1) Hematological system development and function, cell- to-cell signaling and interaction, reproductive system development and function (score=35) (Figure 5.A); 2) Carbohydrate metabolism, cell death, endocrine system disorders (score=33) (Figure 5.B); 3) Carbohydrate metabolism, small molecule biochemistry, cellular development (score=30) (Figure 5.C); 4) Molecular transport, renal and urological disease, cellular function and maintenance (score=28) (Figure 5.D); 5) Lipid metabolism, small molecule biochemistry, molecular transport (score=26) (Figure 5.E). The merged top 3 gene networks (score≥30) are depicted in (Figure 6).

### Diagnostic performance

**Figure 7:**
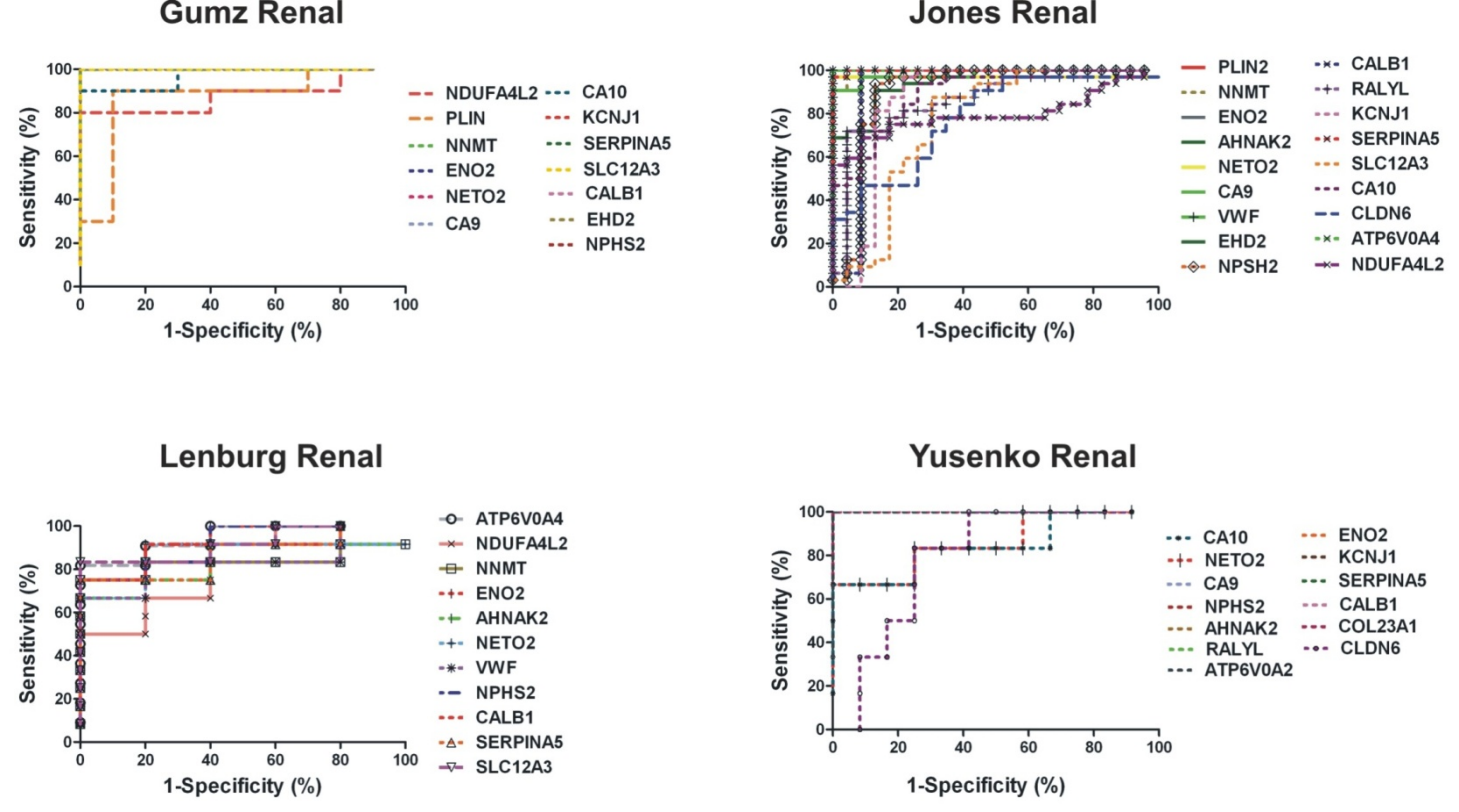
ROC analysis of the top 20 DEGs in ccRCC vs. the normal kidney using each datasets extracted MAS5- calculated signal intensity values Of them, the DEGs with a p<0.01 and an AUC>0.8 were selected as successful distinguishing markers between ccRCC and the normal kidney tissues.In the “Gumz Renal” dataset, NDUFA4L2, PLIN2, NNMT, ENO2, CA9, CA10, KCNJ1, SERPINA5, SLC12A3, CALB1, EHD2 and NPHS2 showed a median AUC=1.00 and p<0.01. In the “Jones Renal” dataset, PLIN2, NNMT, ENO2, AHNAK2, NETO2, CA9, VWF, EHD2, NPHS2, CALB1, RALYL, KCNJ1, SERPINA5, SLC12A3, CA10, CLDN6, ATP6V0A4 and NDUFA4L2 had median AUC=0.969 (p<0.001) and in the “Lenburg Renal” dataset, NDUFA4, NNMT, ENO2, AHNAK2, NETO2, VWF, NPHS2, CALB1, SERPINA5, SLC12A3 and ATP6V0A4 exhibited median AUC=0.90 (p<0.001). In the Yusenko dataset, CA10, NETO2, CA9, NPHS2, AHNAK2, RALYL, ATP6V0A4, ENO2, KCNJ1, SERPINA5, CALB1, COL23A1 and CLDN6 had median AUC values of 1.000 (p<0.01).

A ROC test was performed for the top 20 DEGs in ccRCC using the extracted MAS5-calculated signal intensity values of each gene from each GEO datasets (Table 2). The best discriminatory genes (AUC>0.75 and p<0.05) between ccRCC and the normal tissue in each dataset are depicted in Figure 7.

**Table 2:** Primer pairs used for the amplification of the top up/down-regulated and co-up/down-regulated genes, length of each PCR product and annealing temperature of each pair

Gene	Forward	Reverse	Amplicon size (bp)	Annealing Tm (°C)
Top down-regulated genes				
NDUFA4L2	5′-CCTGAGCCCCAATGACCAATA-3′	5′-TCTGGCCGGTCCTTCTTCA-3′	75	57
PLIN2	5′-ATGGCATCCGTTGCAGTTGAT-3′	5′-GGACATGAGGTCATACGTGGAG-3′	90	57
NNMT	5′-ATATTCTGCCTAGACGGTGTGA-3′	5′-TCAGTGACGACGATCTCCTTAAA-3′	113	60
ENO2	5′-ACAAACAGCGTTACTTAGGCAA-3′	5′-TTCTCAGTCCCATCCAACTCC-3′	148	60
AHNAK2	5′-GTGCAGAAACGGAAGATGACC-3′	5′-GCCTCAGTCGTGTATTCGTAGA-3′	106	57
NETO2	5′-GGACTGGGATTTCGAGCAAAA-3′	5′-AGAGCGCACTATTCCATCAGC-3′	126	56
CA9	5′-TTTGCCAGAGTTGACGAGGC-3′	5′-GCTCATAGGCACTGTTTTCTTCC-3′	97	58
VWF	5′-CCGATGCAGCCTTTTCGGA-3′	5′-TCTGGAAGTCCCCAATAATCGAG-3′	134	60
COL23A1	5′-TCCATCCGAATGTGTCTGCC-3′	5′-GTAGCCATCTCGTCCTGATTG-3′	103	58
EHD2	5′-TCCGCAAACTCAACCCTTTC-3′	5′-TCTCCAGGACCTGATTAGGGA-3′	78	58
NPHS2	5′-ACCAAATCCTCCGGCTTAGG-3′	5′-CAACCTTTACGCAGAACCAGA-3′	106	57
Top up-regulated genes				
CALB1	5′-AACTTTTGTGGATCAGTATGGGC-3′	5′-GGTAATACGTGAGCCAACTCTAC-3′	72	56
RALYL	5′-GAGTGAGCGACATGCAAGAG-3′	5′-GTCAAAGACATAACCGCCAACA-3′	193	57
KCNJ1	5′-CATCCTGGGCCCTGACAAA-3′	5′-AAGCGAGTGACGACCCATTTC-3′	202	58
KNG1	5′-CTAAGACGGTTGGCTCTGACA-3′	5′-TGCCGTGCATTCTCCAGTG-3′	140	58
SERPINA5	5′-AAAGCAAACGAAGGGCAAGATT-3′	5′-CTCTTGGGTGCCTTTGTGGTT-3′	130	58
CLDN8	5′-CTTGGTGGTGTTGGAATGGTG-3′	5′-TCACGCAATTCATCCACAGTC-3′	130	57
SLC12A3	5′-CTCCACCAATGGCAAGGTCAA-3′	5′-GGATGTCGTTAATGGGGTCCA-3′	206	56
CA10	5′-TCATCGTCTGCATATCAGCTCA-3′	5′-GTTCACCAATCCCCAGAAAGAAG-3′	119	56
ATP6V0A4	5′-CTCCCACGGGAAATGATTACC-3′	5′-CGTCTCAAAGAAGTCTTGGGTT-3′	156	60
ACTB	5′-CCAGCACAATGAAGATCAAGATCA-3′	5′-TAGTCCGCCTAGAAGCATTTGC-3′	172	60
RPL13A	5′-CCTGGAGGAGAAGAGGAAAGAGA-3′	5′-TTGAGGACCTCTGTGTATTTGTCAA-3′	101	60
GAPDH	5′-GGAAGGTGAAGGTCGGAGTCA-3′	5′-GTCATTGATGCCAACAATATCCACT-3′	127	60
Co-Up-regulated among four datasets
BTN3A3	5′-AACCACCATTCTTCAGTGGG-3′	5′-GAAGGAAAGCCAGGGAACTT-3′	146	60
Co-up-regulated among three datasets				
PDIA5	5′-AGTGGAGAAAGGAGCCAGC-3′	5′-TGCAGAGGACAGCCATGA-3′	110	60
BHLHE41	5′-GGGACATCTGGAGAAAGCTG-3′	5′-ATCCAAGTCGGACTGAATGG-3′	148	60
SLC12A1	5′-TGAGATTCACGAGCAACTCGC-3′	5′-CCCATCACCGTTAGCAACTCT-3′	76	60
VEGFA	5′-ATGACGAGGGCCTGGAGTGTG-3′	5′-CCTATGTGCTGGCCTTGGTGAG-3′	91	60
CYBB	5′-TCGAAATCTGCTGTCCTTCC-3′	5′-AATCATCCATGCCACCATTT-3′	109	60
ARHGDIB	5′-GACTGGGGTGAAAGTGGATAAAG-3′	5′-TCGTCGGTGAAGAAGGACTTG-3′	150	60
NKG7	5′-TCCAGACCTTCTTCTCCTGG-3′	5′-GCCTTCTGCTCACAAGGTTT-3′	134	60
ATP2B4	5′-CTAGCTTGGTTGCCACACTG-3′	5′-GAGCTTCCTGGATACCGATG-3′	150	60
CAV1	5′-CGAGAAGCAAGTGTACGACG-3′	5′-TCCCTTCTGGTTCTGCAATC-3′	122	60
EGLN3	5′-AGCTTCCTCCTGTCCCTCAT-3′	5′-CTGTTCCATTTCCCGGATAG-3′	118	60
IGFBP3	5′-AACGCTAGTGCCGTCAGC-3′	5′-GACGGGCTCTCCACACTG-3′	113	60
LAIR1	5′-GGCCTAGTGCTCTGCCTG-3′	5′-ACACGAAAGTCACATGGCTC-3′	118	60
NR3C1	5′-TTCCCTGGTCGAACAGTTTT-3′	5′-AGAGTTTGGGAGGTGGTCCT-3′	115	60
PFKP	5′-GTGCGCATGGGTATCTACG-3′	5′-ACTTGCAGGATGCTGGAGAC-3′	125	60
RNASET	5′-GTACTTTGGCAGAAGCCTGG-3′	5′-CCATATACTCTGGCAAGGGC-3′	132	60
Co-down-regulated among four datasets				
TMPRSS2	5′-GGACAGTGTGCACCTCAAAGAC-3′	5′-TCCCACGAGGAAGGTCCC-3′	71	60

### Validation of the DEGs in ccRCC cell lines and a ccRCC patient cohort

**Figure 8:**
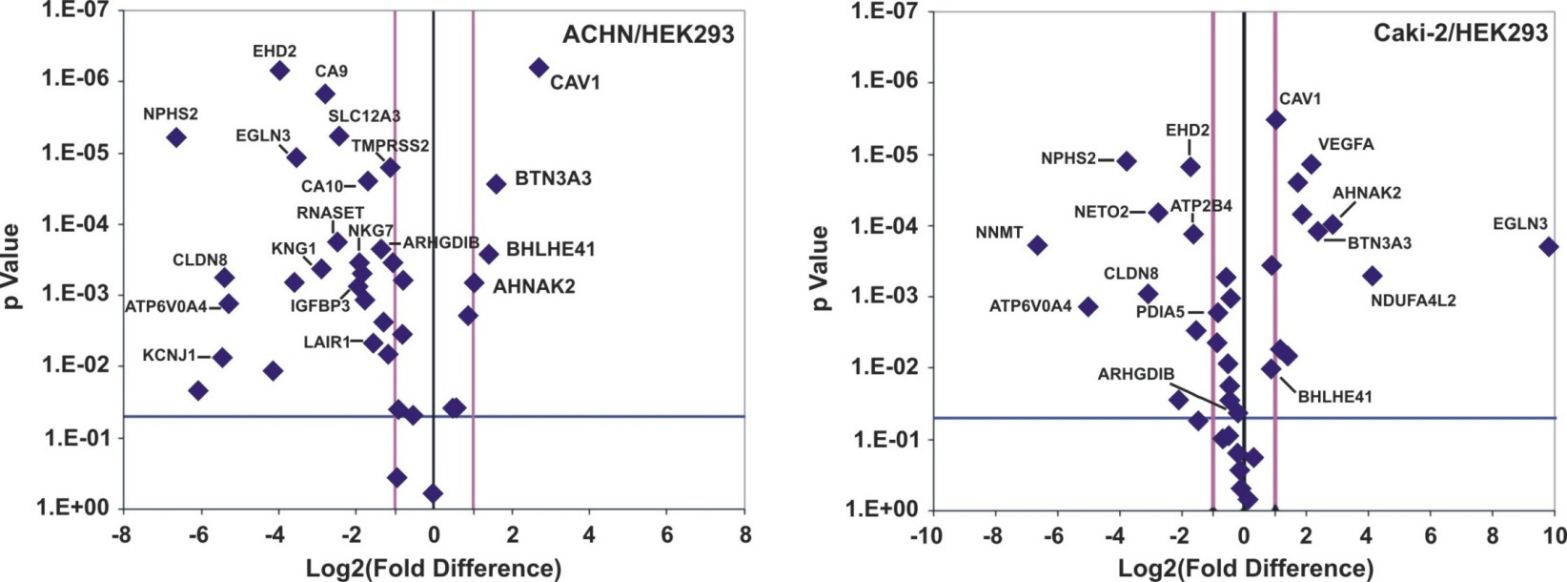
The Volcano-plots depict the DEGs in ACHN and Caki-2 cell lines compared to the HEK-293 cells

In order to validate the DEGs we used two ccRCC cell lines (ACHN and Caki-2) and the HEK-293 cells were used as a control. We also validated the DEGs in a cohort of 10 ccRCC freshly frozen patient samples. The significantly reduced expression levels of *ARHGDIB, NKG7, IGFBP3, LAIR1, RNASET, TMPRSS2 and EGLN3* and the significantly high expression of *AHNAK2* were validated in the ACHN vs. HEK-293 cells. Furthermore, *PDIA5, ARHGDIB and ATP2B4* down-regulation *EGLN3* along with *NDUFA4L2* and *AHNAK2* up-regulation was validated in the Caki-2 vs. HEK-293 cells (Figure 8).

**Figure 9:**
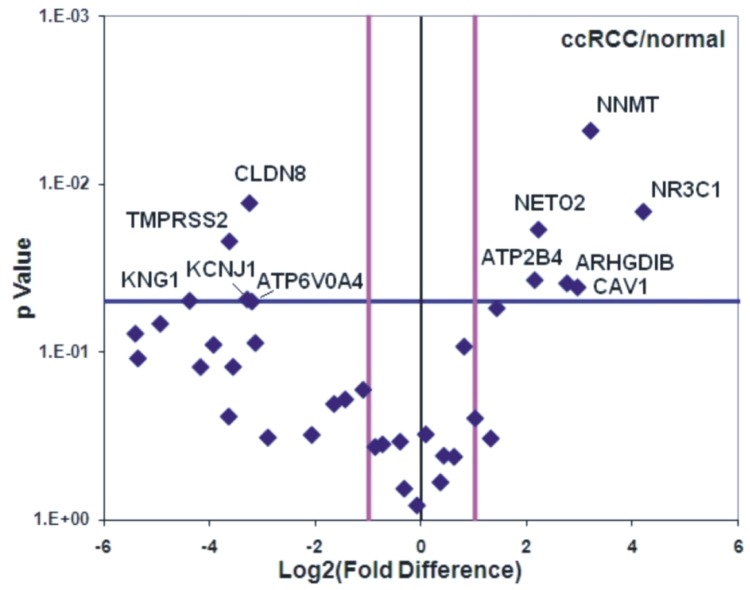
The Volcano-plot depicts the DEGs in a cohort of 10 ccRCC patient samples compared to the adjacent normal kidney samples

**Figure 10:**
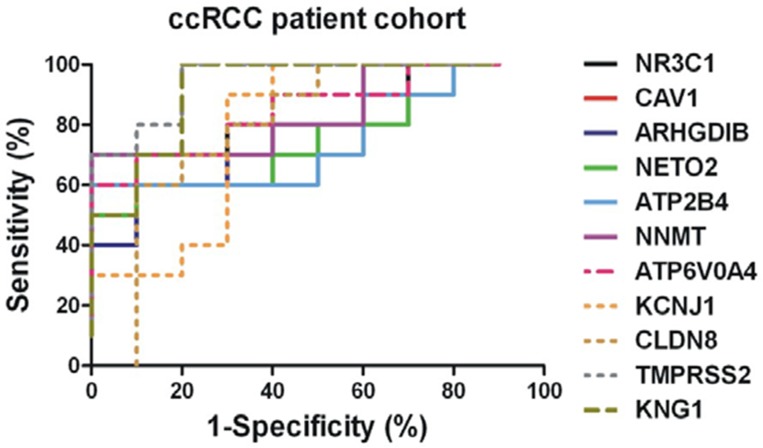
ROC analysis of the validated DEGs in the cohort of the ccRCC patients

In the patient cohort, *ATP6V0A4, KCNJ1, CLDN8, TMPRSS2* and *KNG1* were significantly reduced, whereas *NNMT, NR3C1, CAV1, ARHGDIB, NETO2* and *ATP2B4* mRNA levels were significantly elevated in ccRCC (Figure 9). The normalized expression values of the latter revealed good discriminatory ability between ccRCC and normal kidney (Figure 10 and Table 3).

**Table 3:** Top 10 up- and top 10 down-regulated genes in ccRCC versus the normal kidney tissue Fold change difference and statistical significance are depicted

Top 10 up-regulated molecules	Fold change up-regulation	p-value
NDUFA4L2	53.935	<0.01
PLIN2	27.86	<0.01
NNMT	20.86	<0.01
ENO2	19.973	<0.01
AHNAK2	16.622	<0.01
NETO2	15.808	<0.01
CA9	14.483	<0.01
VWF	13.061	<0.01
COL23A1	12.752	<0.01
EHD2	12.696	<0.01
Top 10 down-regulated molecules	Fold change down-regulation	p-value
ATP6V0A4	−19.699	<0.01
CA10	−21.452	<0.01
SLC12A3	−23.667	<0.01
CLDN8	−27.113	<0.01
SERPINA5	−35.449	<0.01
KNG1	−38.45	<0.01
KCNJ1	−50.79	<0.01
RALYL	−53.576	<0.01
CALB1	−103.68	<0.01
NPHS2	−159.107	<0.01

### Upregulation of NNMT and NR3C1 expression in renal biopsies

**Figure 11:**
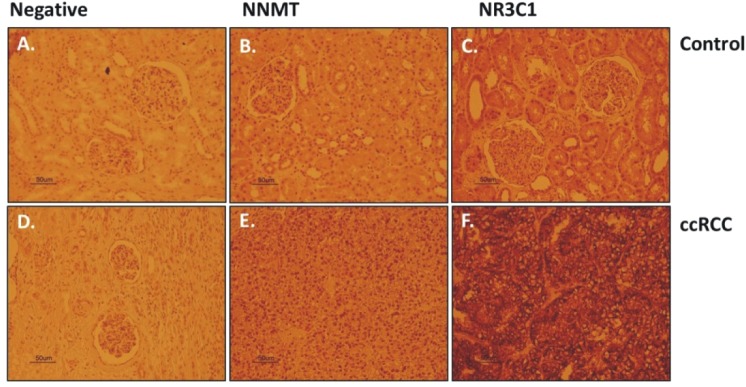
Kidney biopsies from normal kidney (control) and ccRCC patients were stained with anti-NNMT and anti-NR3C1 antibodies In the patients with confirmed ccRCC, serial sections showed stronger NNMT and NR3C1 immunoreactivity as compared to the controls.

Immunohistochemical (IHC) staining of NNMT and NR3C1 proteins was examined in FFPE biopsy samples of 24 confirmed ccRCC patients. Both NNMT and NR3C1 expression was increased in the kidney sections of these patients as compared to biopsies from non-cancerous kidney tissue (Figure 11).

### Further Enrichment analyses

GO enrichment for the top DEGs revealed genes that participate in biological processes such as response to organic substance (adjP=0.0002), response to chemical stimulus (adjP=0.0055), excretion (adjP=0.0067), regulation of epithelial cell proliferation (adjP=0.0067), response to hormone stimulus (adjP=0.0067), response to steroid hormone stimulus (adjP=0.0067), response to oxygen levels (adjP=0.0081), response to endogenous stimulus (adjP=0.0081), response to hypoxia (adjP=0.0081) and epithelial cell proliferation (adjP=0.0081). GO enrichment for the co-upregulated and the co-downregulated genes was also performed (Table S2). Among the most important biological processes of the up-regulated genes, response to hypoxia/oxygen levels (EGLN3, VEGFA, CASP1, FLT1 and CA9) and positive regulation of the vascular endothelial growth factor receptor signaling pathway (VEGFA and FLT1) could be discriminated.

KEGG enrichment was also performed to identify the pathways in which the top DEGs participate (Table S3). Among the co-upregulated genes, CSF2RB, VEGFA, FLT1 and CSF1R participate in the Cytokine- cytokine receptor interaction pathway (adjP=0.0003); VWF, VEGFA and FLT1 are related to the Focal adhesion pathway (adjP=0.0018) and EGLN3 and VEGFA participate in the Renal cell carcinoma pathway (adjP=0.0020). Among the co-downregulated genes, ADH6, ALAD, ALDH6A1, FBP1, MAN1C1, CYP2B6, ABAT, HAO2, ALDOB, INPP5J, PIPOX and ATP6V1B1 participate in Metabolic pathways (adjP=1.78e-08); ADH6, ALDOB and FBP1 are related to Glycolysis/Gluconeogenesis (adjP=0.0003); ALDOB and FBP1 are related to the Fructose and mannose metabolism pathway (adjP=0.0017) and the pentose phosphate pathway (adjP=0.0017). Furthermore, ABAT and ALDH6A1 participate in the Propanoate metabolism pathway (adjP=0.0017) and the Valine, leucine and isoleucine degradation pathway (adjP=0.0023). Other metabolic pathways were also deregulated, such as the Metabolism of xenobiotics by cytochrome P450; the Retinol metabolism and the Drug metabolism - cytochrome P450 pathways (CYP2B6 and ADH6, adjP=0.0034).

Likewise, Wikipathways (Table S4) and Pathway Commons (Table S5) enrichment analysis were performed for the top deregulated genes, as well as for the co-up- and co-downregulated ones. Enrichment analysis for the targets of the co-upregulated and co-downregulated transcription factors was finally performed, using as data source the MSigDB (Table S6). The transcription factors E12 (adjP=1.78e-06), NFAT (adjP=1.50e-05), SP1 (adjP=0.0030) and FOXO4 (adjP=2.28e-05) regulated the highest number of the current 169 co-downregulated genes (12, 8, 9 and 9 genes, respectively), whereas PAX4 (adjP=0.0019) and GATA1 (adjP=0.0009) regulated the highest number of the co-upregulated genes (6 and 4, respectively).

## DISCUSSION

In the present study, we performed a meta-analysis in order to identify deregulated genes in ccRCC compared to the normal kidney and to measure their discriminatory capability between the two tissues. For this purpose, we extracted data from five Oncomine datasets and analyzed them, accordingly. A list of co-deregulated genes among the five microarray datasets was identified and IPA revealed the canonical pathways in which they are implicated, as well as the networks that they form and their associated functions. The deregulated expression pattern of these genes was validated in two ccRCC cell lines and in a cohort of ccRCC patients. We also attempted to gain a better understanding of the molecular pathways/mechanisms involved in ccRCC, through comprehensive bioinformatics analyses.

Knowledge of gene regulatory networks is considered to be of crucial importance for the understanding of diseases such as cancer, as it may lead to new therapeutic approaches. Our investigation revealed that the top 1% of the co-DEGs take part in the Antigen Presentation pathway, the Inositol Metabolism pathway, the Pentose Phosphate pathway, in Glycolysis/Gluconeogenesis, as well as in the Metabolism of Fructose and Mannose. Similar observations corroborating our results were recently reported by others [15-19]. White et al. [15] used three independent algorithms and also showed that the aforementioned pathways are among the top deregulated pathways in metastatic RCC.

In the current study, we found that there was a significant overlap (77 genes) between genes deregulated in ccRCC and those reported to be deregulated in other cancers, suggesting that different cancer types share common pathways. Furthermore, 32 of them play a significant role in renal and urological diseases. The most significant molecular and cellular functions of these co-DEGs involved cell-to-cell signaling, cellular function and maintenance, molecular transport, cellular growth and proliferation.

Our analysis revealed that the antigen presentation pathway was the most altered pathway in ccRCC and BTN3A3 was the most up-regulated gene among all five ccRCC datasets. The butyrophilin (BTN) genes are a group of major histocompatibility complex (MHC)-associated genes that encode type I membrane proteins with 2 extracellular immunoglobulin (Ig) domains and an intracellular B30.2 (PRYSPRY) domain. Autoantibodies that are produced against tumor-associated antigens attract a growing interest for cancer detection, differential diagnostics and prognosis. In line with our analysis, BTN3A3 antigen and its cognate autoantibody was recently suggested as a valuable target for further analysis as potential cancer biomarker [20].

Extensive data in the literature show that cancer cells reprogram their metabolism and shift from aerobic to anaerobic respiration even in the presence of oxygen. This theory was initially proposed by Warburg over 50 years ago [21] and has been recently refreshed [9,22-25]. Reversal of the Warburg effect has also been explored as a novel treatment for cancer [26]. Now considered a hallmark of cancer, metabolic reprogramming of cells results in an unusually high rate of glycolysis and lactate production even in the presence of oxygen. Both our IPA and KEGG enrichments supported that the majority of the deregulated pathways in ccRCC are related to metabolic processes. Glycolysis was among the top deregulated pathways among the five datasets. Our observations are in line with the increasing evidence for the role of altered metabolism in the pathogenesis of renal cancer [2,15,22,27-29]. Our data are also consistent with reports from other cancers, such as metastatic cervical carcinoma and head and neck cancers [30-32]. In these works the authors reported increased glycolysis as measured by high levels of lactate in these tumours. In metastasis, it has been hypothesized that the glycolytic phenotype arises as a result of transient hypoxic episodes that occur while cells travel to distant sites through the bloodstream [33]. Ultimately, cells that are able to perform glycolysis and are resistant to hypoxia, will be selectively favored for survival and growth and result in successful metastasis [33]. Another proposed explanation for the shift to glycolysis is that cancer cells may have damage to their mitochondria through acquired mutations, or may even shut down their mitochondria being the powerhouse of the cell and helps regulate apoptosis. In our meta-analysis, the platelet isoform of phosphofructokinase (PFKP) (ATP D-fructose- 6-phosphate-1-phosphotransferase) was among the most co-upregulated genes among all ccRCC datasets. Of specific note is that our data show that PFKP participates in almost all of the major deregulated canonical pathways in the disease: the pentose phosphate pathway, glycolysis/gluconeogenesis and fructose and mannose metabolism pathway. PFK catalyzes the irreversible conversion of fructose-6-phosphate to fructose-1,6-bisphosphate and is a key regulatory enzyme in glycolysis. It has been shown to be abundantly expressed in human tumors and its expression was linked to long-standing observations concerning the apparent coupling of enhanced glycolysis and cell proliferation [34,35].

A comprehensive understanding of the deregulated metabolic pathways in cancer has much potential for the development of novel therapies. For example, the use of glufosfamide (D-19575), a cytotoxic alkylating agent in which isophosphoramide mustard, the cytotoxic metabolite of ifosfamide, is covalently linked to β-D- glucose [8,36], has been studied for its effectiveness as a treatment for cancers alone or in combination with other treatments [37-39]. In addition, the identification of novel pathways involved in tumorigenesis may allow for the discovery of new compounds that can induce synthetic lethality. After the discovery that RCC tumors, like many others, depend on glycolysis, Chan et al. [40] determined this dependence to be, in part, a result of induction of the glucose transporter 1 (GLUT1). They identified a compound that inhibited the uptake of glucose through GLUT1, which resulted in cancer cell death. Because GLUT1 dependence was observed only in cancer cells lacking the von Hippel-Lindau (VHL) gene, treatment with the GLUT1 inhibiting compound selectively killed cancer cells and had no effect on normal kidney cells. Exploitation of this dependence has also been shown to be promising for the treatment of other cancers.

The data from the present study corroborate that kidney cancer cells manipulate more than one molecular mechanisms and a number of biological pathways to achieve their aggressive phenotype. Renal carcinoma is made up of a number of cancers that occur in the kidney, each having a different histology, following a different clinical course, responding differently to therapy and caused by different genes. Here, we highlight that ccRCC is fundamentally a metabolic disorder. Understanding the mechanisms that lead to altered metabolic pathways in this disease should provide the foundation for the development of novel targeted therapies and development of novel biomarkers.

## MATERIALS AND METHODS

### Data mining and gene expression analysis

The data mining strategy for selecting ccRCC marker genes was based on the Oncomine v4.4.3 cancer microarray platform [14]. Oncomine incorporates more than 628 independent microarray datasets, totaling more than 62,015 microarray experiments and spanning 41 cancer types. It unifies a large compendium of other published cancer microarray data, including Gene Expression Omnibus (GEO) and Stanford Microarray Database (SMD) and uniquely provides differential expression analyses comparing most major types of cancer with their respective normal tissues.

#### Microarray expression datasets

Clear cell Renal Cell Carcinoma was used in the <profile search> function in the Oncomine database to find the available microarray datasets related to the specific cancer type. The analysis type <cancer vs. normal> was then applied to filter those microarray datasets exploring cancer relative to its non-tumor kidney tissue. Five publicly available datasets of gene expression profiles were chosen in this study. These were Gumz Renal [41], Higgins Renal [7], Jones Renal [8], Lenburg Renal [9] and Yusenko Renal [42]. The datasets were carefully selected with the criterion of using the same platform (Affymetrix HU133A and HU133B) (Table 4).

**Table 4:** ROC test for each dataset's top 20 DEGs using their extracted MAS5-calculated signal intensity values

		NDUFA4L2	PLIN2	NNMT	ENO2	AHN AK2	NETO2	CA9	VWF	COL23 A1	EHD2	NPHS2	CALB1	RALYL	KCNJ1	KNG1	SERPINA5	SLC12A3	CA10	CLDN6	ATP6V0A4
Gumz Renal	AUC	0.88	0.87	1.00	1.00	N/A	1.00	1.00	0.56	N/A	1.00	1.00	1.00	N/A	1.00	N/A	1.00	1.00	0.97	0.57	0.65
	Std. Error	0.09	0.09	0.00	0.00	N/A	0.00	0.00	0.14	N/A	0.00	0.00	0.00	N/A	0.00	N/A	0.00	0.00	0.03	0.14	0.13
	95% CI	0.71-1.05	0.69-1.04	1.00-1.00	1.00-1.00	N/A	1.00-1.00	1.00-1.00	0.29-0.83	N/A	1.00-1.00	1.00-1.00	1.00-1.00	N/A	1.00-1.00	N/A	1.00-1.00	1.00-1.00	0.90-1.03	0.29-0.84	0.39-0.90
	p-value	0.00	0.01	0.00	0.00	N/A	0.00	0.00	0.65	N/A	0.00	0.00	0.00	N/A	0.00	N/A	0.00	0.00	0.00	0.60	0.26
Jones Renal	AUC	0.80	1.00	1.00	1.00	1.00	0.97	0.98	1.00	N/A	0.95	0.90	0.92	0.86	0.86	N/A	1.00	0.76	0.89	0.78	1.00
	Std. Error	0.06	0.00	0.01	0.00	0.00	0.03	0.02	0.00	N/A	0.03	0.06	0.06	0.05	0.07	N/A	0.00	0.07	0.04	0.06	0.00
	95% CI	0.67-0.92	1.00-1.00	0.99-1.00	1.00-1.00	1.00-1.00	0.91-1.02	0.95-1.01	1.00-1.00	N/A	0.89-0.99	0.79-1.00	0.80-1.02	0.76-0.97	0.73-0.99	N/A	0.99-1.00	0.61-0.90	0.81-0.97	0.65-0.90	1.00-1.00
	p-value	0.00	<0.0001	<0.0001	<0.0001	<0.0001	<0.0001	<0.0001	<0.0001	N/A	<0.0001	<0.0001	<0.0001	<0.0001	<0.0001	N/A	<0.0001	0.00	<0.0001	0.00	<0.0001
Lenburg Renal	AUC	0.82	0.77	0.83	0.93	0.83	0.87	0.75	0.83	0.80	0.68	0.92	0.90	0.80	0.73	N/A	0.87	0.92	0.53	0.67	0.95
	Std. Error	0.11	0.16	0.10	0.06	0.10	0.09	0.13	0.10	0.11	0.16	0.07	0.08	0.11	0.14	N/A	0.09	0.07	0.14	0.14	0.05
	95% CI	0.59-1.03	0.44-1.08	0.63-1.03	0.81-1.05	0.63-1.03	0.68-1.05	0.50-0.99	0.63-1.03	0.58-1.01	0.36-0.99	0.78-1.05	0.74-1.05	0.58-1.01	0.45-1.00	N/A	0.69-1.04	0.78-1.05	0.25-0.81	0.39-0.93	0.83-1.05
	p-value	0.05	0.09	0.04	0.01	0.04	0.02	0.11	0.04	0.06	0.25	0.01	0.01	0.06	0.14	N/A	0.02	0.01	0.83	0.29	0.01
Yusenko Renal	AUC	0.51	0.67	0.89	1.00	1.00	0.86	1.00	0.61	1.00	0.89	1.00	1.00	1.00	1.00	N/A	1.00	0.78	0.85	0.79	1.00
	Std. Error	0.16	0.27	0.12	0.00	0.00	0.10	0.00	0.16	0.00	0.12	0.00	0.00	0.00	0.00	N/A	0.00	0.11	0.11	0.11	0.00
	95% CI	0.19-0.83	0.13-1.20	0.64-1.13	1.00-1.00	1.00-1.00	0.66-1.05	1.00-1.00	0.29-0.93	1.00-1.00	0.64-1.13	1.00-1.00	1.00-1.00	1.00-1.00	1.00-1.00	N/A	1.00-1.00	0.55-0.99	0.63-1.06	0.58-1.00	1.00-1.00
	p-value	0.93	0.44	0.07	0.02	0.02	0.01	0.02	0.45	0.02	0.07	0.02	0.02	0.02	0.02	N/A	0.02	0.06	0.02	0.05	0.02

#### Gene selection procedure

Concept filters in the Oncomine database were used to identify the differentially expressed genes in ccRCC vs. the normal kidney tissue, using the following parameters: p-value=1E-4; Odds Ratio=2.0 and gene rank=top 1%. Next, a corrected Q value threshold (Q≤0.05) was used to filter and retrieve those DEGs with a high confidence. This filtering approach yielded 53 up- and 53 down-regulated genes from the “Higgins Renal”, 126 up- and 126 down- regulated genes from the “Gumz Renal”, 126 up- and 126 down-regulated genes from the “Jones Renal”, 177 up- and 177 down-regulated genes from the “Lenburg Renal” and 195 up- and 195 down-regulated genes from the “Yusenko Renal” datasets, respectively. Then, the co-DEGs among the five microarray datasets were selected as candidate genes for downstream network analysis and their expression levels was further validated in ccRCC cell lines as well as in a cohort of 10 ccRCC patient samples. The median fold change (±SD) value among the datasets was calculated and scored (Table S1).

### Pathway analysis of the co-deregulated genes

DEGs were investigated for network interrelation by Ingenuity Pathway Analysis, version 7 (IPA; Ingenuity Systems, USA). IPA scans the set of input genes to identify networks by using Ingenuity Pathways Knowledge Base for interactions between identified “Focus Genes.” In this study, the DEGs between ccRCC and normal tissue samples and hypothetical interacting genes stored in the knowledge base in IPA software, were used to generate a set of networks with a maximum network size of 35 genes/proteins. Networks were displayed graphically as genes/gene products (“nodes”) and the biological relationships between the nodes (“edges”). All edges are from canonical information stored in the Ingenuity Pathways Knowledge Base. Networks of these genes were generated by IPA based on their connectivity, each ranked by a score. This score indicates the likelihood of the Focus Genes in a network from Ingenuity's knowledge base being found together due to random chance. It is based on the hypergeometric distribution, calculated with the right- tailed Fisher's Exact Test, and corresponds to the negative log of this p-value. A score of 1.5 was set as the cutoff for identifying gene networks. Furthermore, we used IPA in order to identify the top 1% of the deregulated genes and the top canonical pathways in which they participate. Also, IPA was used to reveal the top molecular and cellular functions, as well as the top biological functions of the co-DEGs. Of these genes, the top 10 deregulated molecules were scored.

### Gene expression validation in ccRCC cell lines

The human ccRCC cell lines Caki-2 and ACHN (kindly provided by Dr Nicoletta Gagliano [43]) were used. Cells were cultured in RPMI-1640 medium supplemented with 10% fetal bovine serum (FBS), 2 mmol/l glutamine, antibiotics (100 U/ml penicillin, 0.1 mg/ml streptomycin) and amphotericin B (2.5 μg/ml). Cells were incubated at 37°C at 5% CO_2_ in 75 cm^2^ flasks. The non-cancerous Human Embryonic Kidney cell line (HEK-293) was used as control. Total RNA was isolated from cells at 80% confluency using the Total RNA isolation NucleoSpin RNA II kit (Macherey-Nagel, Duren) and 400 ng were reverse transcribed to cDNA using the ProtoScript M-MuLV first-strand cDNA synthesis kit (New England Biolabs, Ipswich, MA). Real-time PCRs for the validation of the expression profile of the top or co-DEGs were performed in triplicate 20μl reactions on a ViiA^™^; 7 Real-Time PCR System using SYBR^®^; Green Fast Master Mix (Applied Biosystems). The primer pairs were designed to span at least one intron in order to avoid amplification of the contaminating genomic DNA along with cDNA. Their sequence and the corresponding PCR product sizes are listed in Table 5. Relative mRNA levels were calculated by the ΔCt method [44,45] using ACTB, GAPDH and RPL13A as reference genes. The expression levels of the most deregulated genes were measured in the Caki-2 and ACHN cell lines and compared to the corresponding levels in the HEK-293 cells. The correct size of the PCR products was confirmed by electrophoresis on a 2% agarose gel stained with ethidium bromide. Purity of the amplified products was assessed by melting curve analysis.

**Table 5:** ROC test for the ccRCC patient cohort using the normalized expression (2^-ΔCt) values

	NR3C1	CAV1	ARHGDIB	NETO2	ATP2B4	NNMT	ATP6V0A4	KCNJ1	CLDN8	TMPRSS2	KNG1
AUC	0.79	0.84	0.77	0.75	0.75	0.84	0.85	0.79	0.80	0.95	0.92
Std. Error	0.10	0.09	0.10	0.11	0.11	0.09	0.09	0.11	0.10	0.04	0.06
95% CI	0.58-0.99	0.65-1.02	0.55-0.98	0.52-0.97	0.52-0.97	0.65-1.02	0.67-1.02	0.57-1.00	0.58-1.01	0.86-1.03	0.79-1.04
p-value	0.03	0.01	0.04	0.06	0.06	0.01	0.01	0.02	0.02	<0.0001	0.00

### Gene expression validation in a cohort of ccRCC patient samples

Tissue samples were obtained from kidney specimens from 10 patients with sporadic ccRCC who underwent a radical tumour nephrectomy. Immediately after resection, the samples were stored at −80°C. Histological classification was performed according to the WHO and staging according to the UICC-TNM classification (2002). Nuclear grade was scored according to the Fuhrman classification system [46]. Informed consent was obtained from all patients and the study protocol was approved by the Ethics Committee of the Asklipieio General Hospital, Athens. A matched normal kidney biopsy was also collected from each patient. Total RNA was isolated from ccRCC and normal kidney samples using the TRIzol^®^; Reagent and further purified using the RNeasy minikit (Qiagen, Hilden, Germany). RNA yield and quality were determined with a NanoDrop 1000 Spectrophotometer. cDNA synthesis and qRT-PCR was performed in triplicate 10 μL reaction volumes on a 384-plate format of a ViiA™ 7 Real-Time PCR System using the Fast SYBR Green Master Mix (Applied Biosystems, Foster City, CA), as previously described [47-49].

### Immunohistochemistry (IHC)

Formalin-fixed and paraffin embedded (FFPE) kidney tissue samples from patients with ccRCC were retrieved from the archives of the Asklipieio General Hospital, Athens. All patients in this study underwent a radical tumour nephrectomy. Paraffin sections from each specimen were reviewed by a pathologist, were histologically classified according to the WHO classification and staged according to the UICC-TNM classification (2009). Nuclear grade was scored according to the Fuhrman classification system [46]. Informed consent was obtained from all patients. Kidney specimens were collected between 2007 and 2011. Twenty-two ccRCC and 22 normal kidney tissue (parenchyma) samples were enrolled for IHC. For ccRCC and normal kidney staining experiments, 10μm sections were cut and mount on slides coated with suitable tissue adhesive. Then sections were de-paraffinized using xylene and re- hydrated through washes in graded alcohols. Endogenous peroxidase was neutralized using 0.5% v/v hydrogen peroxide/methanol for 10 minutes. Slides were washed with water. Retrieval was carried out using 0.01M citrate retrieval solution (pH 6.0). Sections were subsequently washed with TBS and blocked for 10 minutes using 3% BSA in PBS. Sections were incubated with primary polyclonal antibodies anti-NNMT (PA5-11143) and anti- NR3C1 (PA1-511A) (NNMT, 1:50 dilution; NR3C1, 1:200 dilution) overnight at 4°C. Slides were then washed and incubated with DAKO REAL EnVision, HRP Rabbit/Mouse (ENV). Subsequently, slides were incubated with 3, 3′-diaminobenzidine (DAB) washed thoroughly in running tap water and counterstained with hematoxylin before being dehydrated and mounted. Haematoxylin and eosin (H&E) staining and IHC for several routinely used ccRCC-specific markers such as AE1/AE3 keratins, Vimentin, Ki67, p53 and S-100 was also performed for all sections.

### Diagnostic performance of the top 20 deregulated genes

We performed a Receiver Operating Characteristic (ROC) curves analysis to evaluate the diagnostic performance of the top 20 deregulated genes in each microarray dataset. The MAS5-calculated Signal intensity values extracted from each dataset were used for the analysis. Sensitivity and specificity scores defining the area under the curve (AUC) were used for each candidate gene in order to discriminate between those individuals with ccRCC and those without the disease [50]. ROC curves were constructed using GraphPad Prism v.5 software (San Diego, CA).

### Further Enrichment Analysis

The DEGs were further explored for the pathways in which they participate, using the WebGestalt web-tool (http://bioinfo.vanderbilt.edu/webgestalt), as previously reported [44,51]. Specifically, we applied Gene Ontology (GO), KEGG pathways, Wikipathways and Pathway Commons Analysis. Since the knowledge of common transcriptional regulatory networks could potentially lead to the key treatment for ccRCC, our attention was also focused on the transcription factors (TFs) that regulate the co-DEGs in ccRCC. We performed Transcription Factor Target Analysis. The hypergeometric test, with Bonferroni correction was used for enrichment evaluation analysis. The R function adjP was used in order to adjust the nominal p values of the large number of categories at the same time. The significance level for the adjusted p-value was set at 0.01 and the number of minimum genes for a category was set at 2.

### Statistical Analysis

Differences in gene expression levels between ccRCC and normal kidney tissues were explored using the paired t-test. Numerical values were expressed as the mean±standard deviation (SD). Statistical significance was set at the 95% confidence level (p<0.05). The statistical package GraphPad Prism v.5 (La Jolla, CA) was used for calculations.

## SUPPLEMENTARY TABLES


