# Supplementary material for: Altered metabolic pathways in clear cell renal cell carcinoma: A meta-analysis and validation study focused on the deregulated genes and their associated networks
Source: Oncoscience. 2014 Jan 3;1(2):117–31. doi: 10.18632/oncoscience.13 (PMC4278286; doi:10.18632/oncoscience.13)
Supplement: Supplementary file 1 [file oncoscience-01-0117-s001.pdf]

Table S1. Genes identified as being differentially expressed in five ccRCC gene expression datasets. Each column represents a different microarray study of ccRCC. In each column, filled boxes indicate that the gene was reported to be differentially expressed in ccRCC vs. the normal kidney tissue. Positive values in the boxes indicate that a gene was reported as induced while negative values indicate that a gene is repressed in ccRCC vs. the normal kidney tissue. Non-filled boxes indicate absence of differential expression for the specific gene. The gene identifier reported is the gene symbols associated with a sequence in the unigene database. Ninety-three and 76 genes were co-up-regulated or co-down-regulated respectively, in ccRCC vs. the normal kidney tissue between at least 2 microarray datasets.

	Gene ID	Fold change (ccRCC vs. normal)					median fold change±SD (ccRCC vs. normal)
		Higgins Renal [7]	Gumz Renal [17]	Jones Renal [8]	Lenburg Renal [9]	Yusenko Renal [18]	
co-up-regulated genes between at least two datasets (n=93)	ABCA1		2.62			3.74	3.18±0.78
	ABCC3	5.15	15.08				10.12±7.02
	AHNAK2		15.04			18.20	16.62±2.23
	ALDOA		2.32		2.63		2.48±0.22
	ALDOC	2.22				5.52	3.87±2.33
	ANXA1		5.35			4.85	5.10±0.35
	ANXA4	3.66	3.66				3.66±0.00
	ARHGDIB	3.91	5.74		3.39		3.92±1.24
	ARPC1B		3.53			4.76	4.15±0.87
	ATP2B4		1.92		1.78	2.49	1.92±0.38
	BHLHE41		11.57	22.23	6.80		11.57±7.90
	BTG1				2.26	2.14	2.20±0.08
	BTN3A3	3.21	3.21		3.70	4.33	3.46±0.53
	C1QB		8.02		7.85		7.94±0.12
	C7orf68				8.58	11.29	9.93±1.92
	CA9		14.48		9.18	17.91	14.48±4.40
	CASP1		4.45		1.44		2.94±2.13
	CAV2		5.72			8.60	7.16±2.04
	CD14		4.13		3.16		3.64±0.69
	CD200		2.80		1.57		2.19±0.87
	CD300A			5.01	1.87	10.65	5.00±4.45
	CHST11				1.70	3.45	2.58±1.24
	CLEC2B	4.15	4.15				4.15±0.00
	COL23A1				9.14	16.36	12.75±5.10
	CSF1R	5.72			2.65		4.19±2.17
	CSF2RB		2.71	2.71	4.67		2.71±1.13
	CYBB	4.14			1.42	5.26	4.14±1.98
	EGLN3		12.10		5.01	12.16	12.10±4.11
	EHD2		22.55		2.84		12.70±13.94
	ENO2		25.79		6.63	19.97	19.97±9.82

	ENPP3				5.08	12.59	8.83±5.30
	ERINC3		1.86			2.30	2.07±0.31
	FAM89B			7.09	1.55		4.32±3.91
	FCER1G		3.25		3.11		3.18±0.097
	FKBP1A				2.99	1.73	2.36±0.89
	FLI1	2.26		6.22			4.24±2.80
	FLT1			12.47		7.03	9.75±3.85
	FN1		7.04		5.26		6.15±1.26
	FXYD5		3.29		2.99		3.14±0.21
	GUK1		1.70			2.34	2.02±0.45
	HLA-DMB				2.48	4.81	3.64±1.64
	HLA-DPA1		3.65		4.43		4.04±0.55
	HLA-DPB1		4.87		3.35		4.10±1.08
	HLA-DRA		5.32		3.77		4.54±1.09
	IFI16		4.45			5.10	4.78±0.46
	IGFBP3		12.33		10.96	10.15	10.96±1.10
	IKZF5	1.55		3.15			2.35±1.13
	LAIR1		2.55		1.96	5.97	2.55±2.16
	LAPTM5		5.59		4.27		4.927±0.929
	LCP2		3.78		1.33		2.56±1.73
	LDHA		16.94		2.32		9.63±10.34
	LIPA		4.12		3.87		3.99±0.18
	LPCAT1		2.76		5.98		4.37±2.27
	LY86		3.44		2.52		2.98±0.65
	MS4A6A		6.06		3.09		4.58±2.09
	NDUFA4L2		53.94	23.34	136.50		53.94±58.53
	NETO2		25.58		6.04		15.81±13.81
	NKG7	5.01	5.01			40.16	5.01±20.29
	NNMT		27.82		13.90		20.86±9.84
	NOL3		14.93		3.12		9.02±8.35
	NR3C1		2.47		2.45	2.01	2.45±0.26
	PDIA5	5.02	5.02			4.07	5.02±0.55
	PDK1		8.18		6.34		7.26±1.30
	PFKP		8.39		5.98	10.13	8.39±2.09
	PLEKHO1	2.62			2.40		2.51±0.15
	PLIN2			47.58		8.14	27.86±27.89
	PLOD3				1.93	2.77	2.35±0.59
	PLSCR1		2.81			2.52	2.66±0.20
	POLH			10.79		2.12	6.45±6.13
	PSMB8	2.77			2.98		2.87±0.15
	PSMB9		4.69		5.04		4.87±0.25

	RHOG				1.59	2.41	2.00±0.58
	RNASET2		10.96		7.33	8.56	8.56±1.84
	SCARB1		5.93			8.93	7.43±2.12
	SLC15A3		12.07		1.76		6.92±7.29
	SLC15A4				3.25	3.47	3.36±0.16
	SLC16A3		7.13		5.87	15.70	7.13±5.35
	SLC38A1				3.58	2.14	2.86±1.02
	SPAG4		11.13			1.31	6.22±6.94
	TAGLN2				2.12	2.76	2.44±0.45
	TAPBP		2.09		2.88		2.48±0.56
	TBXAS1				1.66	3.34	2.50±1.18
	TIMP1	5.52				5.77	5.64±0.17
	TLR2		11.32		1.83		6.57±6.71
	TMCC1				4.62	13.47	9.04±6.26
	TNFSF13B				4.34	4.34	4.34±0.00
	TRDA			4.16		15.30	9.73±7.88
	TSPAN4				1.57	2.21	1.89±0.45
	VEGFA		3.81		3.15	4.75	3.80±0.80
	VIM		3.69		3.16		3.43±0.38
	VWF				14.91	11.21	13.06±2.61
	YWHAH	2.01	2.01				2.01±0.00
	ZNF395	4.57				5.47	5.02±0.64
**co-down-regulated genes between at least two datasets (n=76)**	ABARAPL3		-1.99		-1.40		-1.70±0.41
	ABAT		-2.87		-1.32		-2.10±1.09
	ACOT11		-2.40		-1.00		-1.70±0.98
	ACPP			-11.53		-7.04	-9.29±3.17
	ACSF2		-16.75		-8.35		-12.55±5.94
	ADH6			1.37	-1.68		-0.15±2.15
	AIF1L				-3.21	-6.48	-4.84±2.31
	ALAD		-2.51	-1.38	-3.05		-2.51±0.85
	ALDH6A1		-12.11		-12.24		-12.18±0.09
	ALDOB		-35.72		-1.07		-18.40±24.50
	AMFR	-2.16	-1.61				-1.88±0.39
	ARL4D		-3.61		-1.56		-2.58±1.45
	ATP6V0A4		-19.70		-3.03	-65.84	-19.70±32.54
	ATP6V1B1		-29.00			-2.91	-15.95±18.50
	C1orf226				-1.51	-7.10	-4.30±3.95
	CA10		-15.23			-27.68	-21.45±8.80
	CALB1		-103.68		-29.10	-328.80	-103.68±156.02
	CDKN1C				1.07	1.51	1.29±0.31
	CLDN8		-27.11		-5.66	-180.55	-27.11±95.38

	CLIC5		-1.85		-1.06		-1.45±0.55
	CYP2B6		-1.95		-1.06		-1.50±0.63
	DNAJC4	1.43		1.02			1.23±0.29
	DPEP1		-17.17		-15.06		-16.11±1.49
	ELF5			-1.01		-26.10	-13.55±17.74
	EPB41L4B			-5.13		-1.78	-3.45±2.37
	ERBB4		-1.99		-1.03	1.34	-1.03±1.71
	ESRRG				-2.96	-5.18	-4.07±1.57
	FAM171A1		-2.98			-4.72	-3.85±1.23
	FBP1		-7.97		-9.64		-8.81±1.18
	FGF1				-6.11	-17.47	-11.79±8.03
	GATA3		-8.77		-1.26	-19.52	-8.77±9.18
	GCGR		-16.98	-3.66			-10.32±9.42
	GPC3		-5.76		-2.43		-4.09±2.35
	HADH		-3.47	-2.63			-3.05±0.59
	HAO2		-11.36		-10.27		-10.81±0.77
	IGFBP2				-2.88	-9.58	-6.23±4.73
	INPP5J		-11.01	-4.04			-7.53±4.93
	KCNJ1		-98.81	-2.68	-2.77	-110.65	-50.79±59.09
	KNG1		-74.99		-1.91		-38.45±51.67
	LOC100289119		-10.51		-5.41		-7.96±3.60
	LPPR1		-9.51		-3.05	-1.49	-3.05±4.25
	MAL				-7.29	-21.33	-14.31±9.93
	MAN1C1		-3.15		-1.74		-2.44±0.99
	MECOM	-4.10				-2.76	-3.43±0.95
	MT1G	-8.24			-1.47		-4.86±4.79
	NDUFA4	-2.99	-2.11				-2.55±0.62
	NEDD4L	-1.23				1.05	-0.09±1.61
	NPHS2		-159.11		-9.51	-320.26	-159.10±155.41
	PIPOX		-9.16		-5.90		-7.53±2.30
	PROC		-18.42		-1.92		-10.17±11.67
	PRODH2		-10.51		-2.66		-6.59±5.55
	ProSAPiP1		-5.22		-2.19		-3.70±2.14
	PTH1R		-16.26		-9.28	-8.13	-9.28±4.40
	RALYL		-45.78			-61.37	-53.58±11.02
	RHBG		-12.13		-1.94		-7.04±7.20
	S100A2		-8.40		-12.45		-10.42±2.86
	SERPINA5		-58.72		-12.18		-35.45±32.90
	SFRP1		-19.81	-3.18	-1.02		-3.18±10.28
	SLC12A1				-19.59	-4.06	-11.82±10.98
	SLC12A3		-46.08		-1.26		-23.67±31.69

	SLC15A2		-2.03	-1.89			-1.96±0.10
	SLC19A2		-2.56		-1.81	-2.71	-2.56±0.48
	SLC34A1		-4.46		-1.49		-2.98±2.10
	SLC4A1		-34.00		-2.57		-18.28±22.22
	SLC4A4		-1.89		-1.02		-1.45±0.62
	SLC7A8		-2.90		-1.19		-2.04±1.20
	SORCS1				-1.34	-1.33	-1.34±0.00
	STRA6		-4.15		-1.91		-3.03±1.58
	SUCLG1		-4.70		1.04		-1.83±4.06
	TFAP2A		-1.10		1.01	1.17	1.00±1.26
	TFAP2B			-4.76		1.03	-1.87±4.09
	TFCP2L1		-4.66		-4.02	-16.42	-4.66±6.98
	TMPRSS2		-3.07	-4.64	-1.05	-1.54	-2.30±1.62
	TRIM10			1.23	1.04		1.13±0.14
	TRIM2				1.02	-1.81	-0.40±2.00
	XPNPEP2		1.55		1.75		1.65±0.14

Table S2. Gene Ontology (GO) enrichment for the co-upregulated and the co-downregulated genes. The results for each enriched GO category are listed. For each GO category, the first row lists its sub-root (biological process, molecular function, or cellular component), category name, and corresponding GO ID. The second row lists number of reference genes in the category (C), number of genes in the gene set and also in the category (O), expected number in the category (E), Ratio of enrichment (R), p value from hypergeometric test (rawP), and p value adjusted by the multiple test adjustment (adjP). Finally, genes in the category are listed. For each gene, the table lists the user uploaded ID and value (optional), Entrez ID, Ensembl Gene Stable ID, Gene symbol, and description. Ensembl Gene Stable ID and Entrez Gene ID are linked to the Ensembl and Entrez Gene databases, respectively.

**Gene Ontlogy (GO) for the co-upregulated genes**				
**biological process----response to hypoxia----GO:0001666**				
C=128;O=5;E=0.24;R=20.79;rawP=3.58e-06;adjP=0.0005				
EGLN3	112399	ENSG00000129521	EGLN3	egl nine homolog 3 (C. elegans)
VEGFA	7422	ENSG00000112715	VEGFA	vascular endothelial growth factor A
CASP1	834	ENSG00000137752	CASP1	caspase 1, apoptosis-related cysteine peptidase (interleukin 1, beta, convertase)
FLT1	2321	ENSG00000102755	FLT1	fms-related tyrosine kinase 1 (vascular endothelial growth factor/vascular permeability factor receptor)
CA9	768	ENSG00000107159	CA9	carbonic anhydrase IX
**biological process----response to oxygen levels----GO:0070482**				
C=135;O=5;E=0.25;R=19.71;rawP=4.64e-06;adjP=0.0005				
EGLN3	112399	ENSG00000129521	EGLN3	egl nine homolog 3 (C. elegans)
VEGFA	7422	ENSG00000112715	VEGFA	vascular endothelial growth factor A
CASP1	834	ENSG00000137752	CASP1	caspase 1, apoptosis-related cysteine peptidase (interleukin 1, beta, convertase)
FLT1	2321	ENSG00000102755	FLT1	fms-related tyrosine kinase 1 (vascular endothelial growth factor/vascular permeability factor receptor)
CA9	768	ENSG00000107159	CA9	carbonic anhydrase IX
**biological process----positive regulation of vascular endothelial growth factor receptor signaling pathway----GO:0030949**				
C=7;O=2;E=0.01;R=152.06;rawP=7.10e-05;adjP=0.0032				
VEGFA	7422	ENSG00000112715	VEGFA	vascular endothelial growth factor A

FLT1	2321	ENSG00000102755	FLT1	fms-related tyrosine kinase 1 (vascular endothelial growth factor/vascular permeability factor receptor)
**biological process----regulation of smooth muscle cell proliferation----GO:0048660**				
C=40;O=3;E=0.08;R=39.92;rawP=5.58e-05;adjP=0.0032				
VEGFA	7422	ENSG00000112715	VEGFA	vascular endothelial growth factor A
FLT1	2321	ENSG00000102755	FLT1	fms-related tyrosine kinase 1 (vascular endothelial growth factor/vascular permeability factor receptor)
IGFBP3	3486	ENSG00000146674	IGFBP3	insulin-like growth factor binding protein 3
**biological process----smooth muscle cell proliferation----GO:0048659**				
C=42;O=3;E=0.08;R=38.02;rawP=6.47e-05;adjP=0.0032				
VEGFA	7422	ENSG00000112715	VEGFA	vascular endothelial growth factor A
FLT1	2321	ENSG00000102755	FLT1	fms-related tyrosine kinase 1 (vascular endothelial growth factor/vascular permeability factor receptor)
IGFBP3	3486	ENSG00000146674	IGFBP3	insulin-like growth factor binding protein 3
**biological process----regulation of vascular endothelial growth factor receptor signaling pathway----GO:0030947**				
C=9;O=2;E=0.02;R=118.27;rawP=0.0001;adjP=0.0038				
VEGFA	7422	ENSG00000112715	VEGFA	vascular endothelial growth factor A
FLT1	2321	ENSG00000102755	FLT1	fms-related tyrosine kinase 1 (vascular endothelial growth factor/vascular permeability factor receptor)
**biological process----muscle cell proliferation----GO:0033002**				
C=56;O=3;E=0.11;R=28.51;rawP=0.0002;adjP=0.0065				
VEGFA	7422	ENSG00000112715	VEGFA	vascular endothelial growth factor A
FLT1	2321	ENSG00000102755	FLT1	fms-related tyrosine kinase 1 (vascular endothelial growth factor/vascular permeability factor receptor)

IGFBP3	3486	ENSG00000146674	IGFBP3	insulin-like growth factor binding protein 3
**molecular function----protein dimerization activity----GO:0046983**				
C=514;O=6;E=0.94;R=6.41;rawP=0.0003;adjP=0.0089				
VWF	7450	ENSG00000110799	VWF	von Willebrand factor
C1QB	713	ENSG00000173369	C1QB	complement component 1, q subcomponent, B chain
VEGFA	7422	ENSG00000112715	VEGFA	vascular endothelial growth factor A
ENO2	2026	ENSG00000111674	ENO2	enolase 2 (gamma, neuronal)
CYBB	1536	ENSG00000165168	CYBB	cytochrome b-245, beta polypeptide
CSF1R	1436	ENSG00000182578	CSF1R	colony stimulating factor 1 receptor
**molecular function----protein homodimerization activity----GO:0042803**				
C=318;O=5;E=0.58;R=8.63;rawP=0.0002;adjP=0.0089				
VWF	7450	ENSG00000110799	VWF	von Willebrand factor
C1QB	713	ENSG00000173369	C1QB	complement component 1, q subcomponent, B chain
VEGFA	7422	ENSG00000112715	VEGFA	vascular endothelial growth factor A
ENO2	2026	ENSG00000111674	ENO2	enolase 2 (gamma, neuronal)
CSF1R	1436	ENSG00000182578	CSF1R	colony stimulating factor 1 receptor
**Gene Ontlogy for the co-downregulated genes**				
**biological process----response to organic substance----GO:0010033**				
C=686;O=10;E=1.96;R=5.11;rawP=1.68e-05;adjP=0.0031				
ADH6	130	ENSG00000172955	ADH6	alcohol dehydrogenase 6 (class V)
FBP1	2203	ENSG00000165140	FBP1	fructose-1,6-bisphosphatase 1
DNAJC4	3338	ENSG00000110011	DNAJC4	DnaJ (Hsp40) homolog, subfamily C, member 4
AMFR	267	ENSG00000159461	AMFR	autocrine motility factor receptor
GCGR	2642	NULL	GCGR	glucagon receptor
GATA3	2625	ENSG00000107485	GATA3	GATA binding protein 3
IGFBP2	3485	ENSG00000115457	IGFBP2	insulin-like growth factor binding protein 2, 36kDa
ABAT	18	ENSG00000183044	ABAT	4-aminobutyrate aminotransferase
ALDOB	229	ENSG00000136872	ALDOB	aldolase B, fructose-bisphosphate

ERBB4	2066	ENSG00000178568	ERBB4	v-erb-a erythroblastic leukemia viral oncogene homolog 4 (avian)
**biological process----excretion----GO:0007588**				
C=60;O=4;E=0.17;R=23.37;rawP=2.48e-05;adjP=0.0031				
KNG1	3827	ENSG00000113889	KNG1	kininogen 1
ATP6V1B1	525	ENSG00000116039	ATP6V1B1	ATPase, H+ transporting, lysosomal 56/58kDa, V1 subunit B1
KCNJ1	3758	ENSG00000151704	KCNJ1	potassium inwardly-rectifying channel, subfamily J, member 1
NPHS2	7827	ENSG00000116218	NPHS2	nephrosis 2, idiopathic, steroid-resistant (podocin)

Table S3. KEGG pathways enrichment for the top DEGs. The enriched KEGG pathways and the statistics for the enrichment of the pathway are listed. The statistice column lists the number of reference genes in the category (C), number of genes in the gene set and also in the category (O), expected number in the category (E), Ratio of enrichment (R), p value from hypergeometric test (rawP), and p value adjusted by the multiple test adjustment (adjP).

**KEGG enrichment for the top DEGs**	
**Enriched KEGG pathways**	**statistics for the enrichment of the pathway**
Metabolic pathways	C=1104;O=14;E=1.66;R=8.46;rawP=8.62e-10;adjP=1.81e-08
Complement and coagulation cascades	C=69;O=4;E=0.10;R=38.65;rawP=3.72e-06;adjP=3.91e-05
Glycolysis / Gluconeogenesis	C=62;O=3;E=0.09;R=32.26;rawP=0.0001;adjP=0.0007
Pentose phosphate pathway	C=27;O=2;E=0.04;R=49.39;rawP=0.0008;adjP=0.0034
Cytokine-cytokine receptor interaction	C=267;O=4;E=0.40;R=9.99;rawP=0.0007;adjP=0.0034
Fructose and mannose metabolism	C=34;O=2;E=0.05;R=39.22;rawP=0.0012;adjP=0.0036
Propanoate metabolism	C=33;O=2;E=0.05;R=40.41;rawP=0.0011;adjP=0.0036
Pathways in cancer	C=330;O=4;E=0.49;R=8.08;rawP=0.0016;adjP=0.0042
Valine, leucine and isoleucine degradation	C=44;O=2;E=0.07;R=30.31;rawP=0.0020;adjP=0.0047
Inositol phosphate metabolism	C=54;O=2;E=0.08;R=24.69;rawP=0.0030;adjP=0.0057
Endocytosis	C=187;O=3;E=0.28;R=10.70;rawP=0.0028;adjP=0.0057
Focal adhesion	C=201;O=3;E=0.30;R=9.95;rawP=0.0035;adjP=0.0061
Retinol metabolism	C=64;O=2;E=0.10;R=20.84;rawP=0.0042;adjP=0.0068
Metabolism of xenobiotics by cytochrome P450	C=70;O=2;E=0.10;R=19.05;rawP=0.0050;adjP=0.0074
Drug metabolism - cytochrome P450	C=72;O=2;E=0.11;R=18.52;rawP=0.0053;adjP=0.0074
Neuroactive ligand-receptor interaction	C=256;O=3;E=0.38;R=7.81;rawP=0.0068;adjP=0.0089
**KEGG enrichment for the co-upregulated genes**	
**Enriched KEGG pathways**	**statistics for the enrichment of the pathway**
Cytokine-cytokine receptor interaction	C=267;O=4;E=0.18;R=21.91;rawP=3.26e-05;adjP=0.0003
Focal adhesion	C=201;O=3;E=0.14;R=21.83;rawP=0.0004;adjP=0.0018
Complement and coagulation cascades	C=69;O=2;E=0.05;R=42.39;rawP=0.0010;adjP=0.0020
Renal cell carcinoma	C=70;O=2;E=0.05;R=41.79;rawP=0.0011;adjP=0.0020
Glycolysis / Gluconeogenesis	C=62;O=2;E=0.04;R=47.18;rawP=0.0008;adjP=0.0020
Pathways in cancer	C=330;O=3;E=0.23;R=13.30;rawP=0.0015;adjP=0.0022
Neurotrophin signaling pathway	C=126;O=2;E=0.09;R=23.22;rawP=0.0034;adjP=0.0044
Metabolic pathways	C=1104;O=4;E=0.75;R=5.30;rawP=0.0065;adjP=0.0073
Endocytosis	C=187;O=2;E=0.13;R=15.64;rawP=0.0073;adjP=0.0073
**KEGG enrichment for the co-downregulated genes**	
**Enriched KEGG pathways**	**statistics for the enrichment of the pathway**
Metabolic pathways	C=1104;O=12;E=1.17;R=10.27;rawP=1.27e-09;adjP=1.78e-08
Glycolysis / Gluconeogenesis	C=62;O=3;E=0.07;R=45.70;rawP=4.03e-05;adjP=0.0003

Fructose and mannose metabolism	C=34;O=2;E=0.04;R=55.56;rawP=0.0006;adjP=0.0017
Pentose phosphate pathway	C=27;O=2;E=0.03;R=69.97;rawP=0.0004;adjP=0.0017
Propanoate metabolism	C=33;O=2;E=0.03;R=57.25;rawP=0.0006;adjP=0.0017
Valine, leucine and isoleucine degradation	C=44;O=2;E=0.05;R=42.93;rawP=0.0010;adjP=0.0023
Inositol phosphate metabolism	C=54;O=2;E=0.06;R=34.98;rawP=0.0015;adjP=0.0030
Complement and coagulation cascades	C=69;O=2;E=0.07;R=27.38;rawP=0.0025;adjP=0.0034
Metabolism of xenobiotics by cytochrome P450	C=70;O=2;E=0.07;R=26.99;rawP=0.0025;adjP=0.0034
Retinol metabolism	C=64;O=2;E=0.07;R=29.52;rawP=0.0021;adjP=0.0034
Drug metabolism - cytochrome P450	C=72;O=2;E=0.08;R=26.24;rawP=0.0027;adjP=0.0034

Table S4. Wikipathways enrichment for the top DEGs, the co-upregulated and the co-downregulated genes, respectively. The results for each enriched gene set are listed. For each gene set, the first row lists the gene set name. The second row lists number of reference genes in the category (C), number of genes in the gene set and also in the category (O), expected number in the category (E), Ratio of enrichment (R), p value from hypergeometric test (rawP), and p value adjusted by the multiple test adjustment (adjP).

**Wikipathways enrichment for the top DEGS**	
**Enriched Wiki pathways**	**statistics for the enrichment of the pathway**
Complement and Coagulation Cascades KEGG	C=51;O=4;E=0.08;R=52.29;rawP=1.10e-06;adjP=1.32e-05
Endochondral Ossification	C=65;O=3;E=0.10;R=30.77;rawP=0.0001;adjP=0.0006
GPCRs, Class B Secretin-like	C=23;O=2;E=0.03;R=57.98;rawP=0.0005;adjP=0.0020
Androgen Receptor Signaling Pathway	C=115;O=3;E=0.17;R=17.39;rawP=0.0007;adjP=0.0021
Myometrial Relaxation and Contraction Pathways	C=156;O=3;E=0.23;R=12.82;rawP=0.0017;adjP=0.0040
Glycolysis and Gluconeogenesis	C=44;O=2;E=0.07;R=30.31;rawP=0.0020;adjP=0.0040
Id Signaling Pathway	C=51;O=2;E=0.08;R=26.15;rawP=0.0027;adjP=0.0046
SIDS Susceptibility Pathways	C=66;O=2;E=0.10;R=20.20;rawP=0.0045;adjP=0.0067
Alpha6-Beta4 Integrin Signaling Pathway	C=71;O=2;E=0.11;R=18.78;rawP=0.0052;adjP=0.0069
**Wikipathways enrichment for co-upregulated genes**	
**Enriched Wiki pathways**	**statistics for the enrichment of the pathway**
Androgen Receptor Signaling Pathway	C=115;O=3;E=0.08;R=38.15;rawP=6.78e-05;adjP=0.0005
Id Signaling Pathway	C=51;O=2;E=0.03;R=57.35;rawP=0.0006;adjP=0.0010
Glycolysis and Gluconeogenesis	C=44;O=2;E=0.03;R=66.48;rawP=0.0004;adjP=0.0010
Complement and Coagulation Cascades KEGG	C=51;O=2;E=0.03;R=57.35;rawP=0.0006;adjP=0.0010
Alpha6-Beta4 Integrin Signaling Pathway	C=71;O=2;E=0.05;R=41.20;rawP=0.0011;adjP=0.0015
Myometrial Relaxation and Contraction Pathways	C=156;O=2;E=0.11;R=18.75;rawP=0.0051;adjP=0.0060
Focal Adhesion	C=185;O=2;E=0.13;R=15.81;rawP=0.0071;adjP=0.0071
**Wikipathways enrichment for co-downregulated genes**	
**Enriched Wiki pathways**	**statistics for the enrichment of the pathway**
GPCRs, Class B Secretin-like	C=23;O=2;E=0.02;R=82.14;rawP=0.0003;adjP=0.0012
Glycolysis and Gluconeogenesis	C=44;O=2;E=0.05;R=42.93;rawP=0.0010;adjP=0.0019
Complement and Coagulation Cascades KEGG	C=51;O=2;E=0.05;R=37.04;rawP=0.0014;adjP=0.0019
Endochondral Ossification	C=65;O=2;E=0.07;R=29.06;rawP=0.0022;adjP=0.0022

Table S5. Pathway Commons enrichment for the top DEGs, the co-upregulated and the co-downregulated genes. The results for each enriched gene set are listed. For each gene set, the first row lists the gene set name. The second row lists number of reference genes in the category (C), number of genes in the gene set and also in the category (O), expected number in the category (E), Ratio of enrichment (R), p value from hypergeometric test (rawP), and p value adjusted by the multiple test adjustment (adjP).

**Pathway Commons enrichment for the top DEGs**	
**Enriched Gene Sets**	**statistics for the enrichment of the pathway**
Caspase cascade in apoptosis	C=47;O=3;E=0.07;R=42.56;rawP=4.99e-05;adjP=0.0021
Regulation of IGF Activity by IGFBP	C=14;O=2;E=0.02;R=95.25;rawP=0.0002;adjP=0.0043
Glypican pathway	C=436;O=5;E=0.65;R=7.65;rawP=0.0005;adjP=0.0043
TNF receptor signaling pathway	C=246;O=4;E=0.37;R=10.84;rawP=0.0005;adjP=0.0043
S1P3 pathway	C=19;O=2;E=0.03;R=70.18;rawP=0.0004;adjP=0.0043
VEGFR1 specific signals	C=25;O=2;E=0.04;R=53.34;rawP=0.0007;adjP=0.0049
Biological oxidations	C=118;O=3;E=0.18;R=16.95;rawP=0.0008;adjP=0.0049
Intrinsic Pathway	C=32;O=2;E=0.05;R=41.67;rawP=0.0011;adjP=0.0053
TRAIL signaling pathway	C=297;O=4;E=0.45;R=8.98;rawP=0.0011;adjP=0.0053
Formation of Fibrin Clot (Clotting Cascade)	C=35;O=2;E=0.05;R=38.10;rawP=0.0013;adjP=0.0056
FOXA2 and FOXA3 transcription factor networks	C=39;O=2;E=0.06;R=34.19;rawP=0.0016;adjP=0.0063
Glypican 1 network	C=404;O=4;E=0.61;R=6.60;rawP=0.0032;adjP=0.0076
Response to elevated platelet cytosolic Ca++	C=56;O=2;E=0.08;R=23.81;rawP=0.0032;adjP=0.0076
Exocytosis of Alpha granule	C=46;O=2;E=0.07;R=28.99;rawP=0.0022;adjP=0.0076
Platelet degranulation	C=48;O=2;E=0.07;R=27.78;rawP=0.0024;adjP=0.0076
Signaling by GPCR	C=195;O=3;E=0.29;R=10.26;rawP=0.0032;adjP=0.0076
Class B/2 (Secretin family receptors)	C=52;O=2;E=0.08;R=25.64;rawP=0.0028;adjP=0.0076
Alpha6Beta4Integrin	C=50;O=2;E=0.07;R=26.67;rawP=0.0026;adjP=0.0076
HIF-1-alpha transcription factor network	C=64;O=2;E=0.10;R=20.84;rawP=0.0042;adjP=0.0086
Signaling events mediated by VEGFR1 and VEGFR2	C=63;O=2;E=0.09;R=21.17;rawP=0.0041;adjP=0.0086
Integrins in angiogenesis	C=63;O=2;E=0.09;R=21.17;rawP=0.0041;adjP=0.0086
Innate Immunity Signaling	C=66;O=2;E=0.10;R=20.20;rawP=0.0045;adjP=0.0088
Hypoxic and oxygen homeostasis regulation of HIF-1-alpha	C=77;O=2;E=0.12;R=17.32;rawP=0.0060;adjP=0.0099
FOXA transcription factor networks	C=73;O=2;E=0.11;R=18.27;rawP=0.0054;adjP=0.0099
Syndecan-2-mediated signaling events	C=76;O=2;E=0.11;R=17.55;rawP=0.0059;adjP=0.0099
Platelet Activation	C=74;O=2;E=0.11;R=18.02;rawP=0.0056;adjP=0.0099
**Pathway Commons enrichment for the co-upregulated genes**	
HIF-1-alpha transcription factor network	C=64;O=4;E=0.04;R=91.41;rawP=1.10e-07;adjP=1.76e-06
Hypoxic and oxygen homeostasis regulation of HIF-1-alpha	C=77;O=4;E=0.05;R=75.98;rawP=2.34e-07;adjP=1.87e-06
Caspase cascade in apoptosis	C=47;O=3;E=0.03;R=93.35;rawP=4.60e-

	06;adjP=2.45e-05
S1P3 pathway	C=19;O=2;E=0.01;R=153.95;rawP=7.68e-05;adjP=0.0003
VEGFR1 specific signals	C=25;O=2;E=0.02;R=117.00;rawP=0.0001;adjP=0.0003
Alpha6Beta4Integrin	C=50;O=2;E=0.03;R=58.50;rawP=0.0005;adjP=0.0013
Innate Immunity Signaling	C=66;O=2;E=0.05;R=44.32;rawP=0.0009;adjP=0.0014
TNF receptor signaling pathway	C=246;O=3;E=0.17;R=17.84;rawP=0.0006;adjP=0.0014
Signaling events mediated by VEGFR1 and VEGFR2	C=63;O=2;E=0.04;R=46.43;rawP=0.0009;adjP=0.0014
Integrins in angiogenesis	C=63;O=2;E=0.04;R=46.43;rawP=0.0009;adjP=0.0014
TRAIL signaling pathway	C=297;O=3;E=0.20;R=14.77;rawP=0.0011;adjP=0.0016
Sphingosine 1-phosphate (S1P) pathway	C=113;O=2;E=0.08;R=25.89;rawP=0.0027;adjP=0.0033
Glypican 1 network	C=404;O=3;E=0.28;R=10.86;rawP=0.0026;adjP=0.0033
Glypican pathway	C=436;O=3;E=0.30;R=10.06;rawP=0.0033;adjP=0.0038
Signaling in Immune system	C=211;O=2;E=0.14;R=13.86;rawP=0.0092;adjP=0.0098
**Pathway Commons enrichment for the co-downregulated genes**	
Class B/2 (Secretin family receptors)	C=52;O=2;E=0.06;R=36.33;rawP=0.0014;adjP=0.0091
Signaling by GPCR	C=195;O=3;E=0.21;R=14.53;rawP=0.0012;adjP=0.0091

Table S6. Transcription Factor targets enrichment. The results for each enriched gene set are listed in this table. For each gene set, the first row lists the gene set name. The second row lists number of reference genes in the category (C), number of genes in the gene set and also in the category (O), expected number in the category (E), Ratio of enrichment (R), p value from hypergeometric test (rawP), and p value adjusted by the multiple test adjustment (adjP).

Enrichment TFBM analysis for the co-downregulated genes	
**Enriched Transcription Factors**	**statistics for the enrichment of the pathway**
hsa_CAGGTG_V$E12_Q6	C=1825;O=12;E=1.93;R=6.21;rawP=3.16e-07;adjP=3.25e-05
hsa_V$NFY_C	C=181;O=4;E=0.19;R=20.87;rawP=4.17e-05;adjP=0.0012
hsa_V$ALPHACP1_01	C=186;O=4;E=0.20;R=20.31;rawP=4.63e-05;adjP=0.0012
hsa_TTGTTT_V$FOXO4_01	C=1514;O=9;E=1.60;R=5.61;rawP=2.61e-05;adjP=0.0012
hsa_TGGAAA_V$NFAT_Q4_01	C=1408;O=8;E=1.49;R=5.37;rawP=0.0001;adjP=0.0021
hsa_YATTNATC_UNKNOWN	C=277;O=4;E=0.29;R=13.64;rawP=0.0002;adjP=0.0034
hsa_CTTTGA_V$LEF1_Q2	C=914;O=6;E=0.97;R=6.20;rawP=0.0004;adjP=0.0052
hsa_GGGCGGR_V$SP1_Q6	C=2138;O=9;E=2.26;R=3.98;rawP=0.0004;adjP=0.0052
hsa_AACTTT_UNKNOWN	C=1446;O=7;E=1.53;R=4.57;rawP=0.0008;adjP=0.0081
hsa_V$MYOD_01	C=187;O=3;E=0.20;R=15.15;rawP=0.0010;adjP=0.0081
hsa_V$CEBPA_01	C=189;O=3;E=0.20;R=14.99;rawP=0.0011;adjP=0.0081
hsa_V$NFY_Q6	C=188;O=3;E=0.20;R=15.07;rawP=0.0011;adjP=0.0081
hsa_V$NFY_01	C=180;O=3;E=0.19;R=15.74;rawP=0.0009;adjP=0.0081
hsa_RTAAACA_V$FREAC2_01	C=704;O=5;E=0.75;R=6.71;rawP=0.0009;adjP=0.0081
hsa_V$PAX2_02	C=197;O=3;E=0.21;R=14.38;rawP=0.0012;adjP=0.0082
hsa_V$ZIC1_01	C=201;O=3;E=0.21;R=14.10;rawP=0.0013;adjP=0.0084
hsa_V$CEBP_Q2_01	C=207;O=3;E=0.22;R=13.69;rawP=0.0014;adjP=0.0085
hsa_V$E2F_01	C=55;O=2;E=0.06;R=34.35;rawP=0.0016;adjP=0.0087
hsa_V$OCT1_06	C=217;O=3;E=0.23;R=13.06;rawP=0.0016;adjP=0.0087
hsa_GGGNRMNNYCAT_UNKNOWN	C=60;O=2;E=0.06;R=31.49;rawP=0.0019;adjP=0.0093
hsa_GATTGGY_V$NFY_Q6_01	C=837;O=5;E=0.89;R=5.64;rawP=0.0019;adjP=0.0093
hsa_GGGAGGRR_V$MAZ_Q6	C=1718;O=7;E=1.82;R=3.85;rawP=0.0021;adjP=0.0098
**Enrichment TFBM analysis for the co-upregulated genes**	
**Enriched Transcription Factors**	**statistics for the enrichment of the pathway**
hsa_V$GATA1_05	C=212;O=4;E=0.14;R=27.60;rawP=1.32e-05;adjP=0.0009
hsa_GGGTGGRR_V$PAX4_03	C=1004;O=6;E=0.69;R=8.74;rawP=5.33e-05;adjP=0.0019
hsa_GATAAGR_V$GATA_C	C=234;O=3;E=0.16;R=18.75;rawP=0.0005;adjP=0.0029
hsa_GGGYGTGNY_UNKNOWN	C=490;O=4;E=0.34;R=11.94;rawP=0.0003;adjP=0.0029
hsa_V$RREB1_01	C=164;O=3;E=0.11;R=26.75;rawP=0.0002;adjP=0.0029

hsa_V$AML_Q6	C=197;O=3;E=0.13;R=22.27;rawP=0.0003;adjP=0.0029
hsa_V$AP1_Q2_01	C=221;O=3;E=0.15;R=19.85;rawP=0.0005;adjP=0.0029
hsa_V$GATA1_04	C=194;O=3;E=0.13;R=22.62;rawP=0.0003;adjP=0.0029
hsa_V$AP1FJ_Q2	C=224;O=3;E=0.15;R=19.59;rawP=0.0005;adjP=0.0029
hsa_V$AP1_Q2	C=217;O=3;E=0.15;R=20.22;rawP=0.0004;adjP=0.0029
hsa_V$IRF_Q6	C=175;O=3;E=0.12;R=25.07;rawP=0.0002;adjP=0.0029
hsa_V$HOXA4_Q2	C=205;O=3;E=0.14;R=21.40;rawP=0.0004;adjP=0.0029
